# Impairment of mitochondrial quality control exacerbates diabetes-related atrial fibrillation by cGAS-STING signaling pathway and cardiomyocyte-macrophage crosstalk

**DOI:** 10.7150/thno.124140

**Published:** 2026-01-01

**Authors:** Shan Meng, Jinfeng Duan, Jikai Zhao, Zijun Zhou, Boxuan Sun, Yinli Xu, Tao Huang, Tao Hong, Xin Chen, Tong Su, Liming Yu, Huishan Wang

**Affiliations:** 1 State Key Laboratory of Frigid Zone Cardiovascular Disease, Department of Cardiovascular Surgery, General Hospital of Northern Theater Command, 83 Wenhua Road, Shenyang, Liaoning 110016, P. R. China.; 2 Department of Anaesthesia, The Sixth Affiliated Hospital, Sun Yat-sen University, Guangzhou, Guangdong 510000, P. R. China.; 3 Postgraduate College, China Medical University, Shenyang, Liaoning 110122, P. R. China.; 4 Pediatric Surgery Ward, Fuwai Hospital Chinese Academy of Medical Sciences, ShenZhen 518000, P. R. China.; 5 College of Medicine and Biological Information Engineering, Northeastern University, Shenyang, Liaoning 110167, P. R. China.

**Keywords:** cGAS-STING signaling pathway, Atrial fibrillation, Type 2 diabetes, Mitochondrial quality control, Atrial myocytes-macrophage crosstalk.

## Abstract

**Aims:** Type 2 diabetes mellitus (T2DM) significantly elevates the likelihood of atrial fibrillation (AF); However, the precise mechanisms remain incompletely elucidated. Mitochondrial dysfunction is a hallmark of diabetic cardiomyopathy, and recent evidence suggests that activation of the cGAS-STING signaling pathway may contribute to metabolic inflammation in the atria. This study aims to investigate the role of mitochondrial DNA (mtDNA)-mediated cGAS-STING activation in promoting diabetes-associated atrial fibrillation (AF) through cardiomyocyte-macrophage crosstalk.

**Methods and results:** Using a high-fat diet combined with streptozotocin through intraperitoneal injection, we induced a diabetic mouse model. We observed increased AF inducibility, oxidative stress, and mitochondrial ultrastructural abnormalities, along with elevated expression of STING pathway components and pro-inflammatory cytokines in atrial tissue. RNA sequencing and histological analyses confirmed dysregulation of mitochondrial quality control (MQC), including impaired mitophagy, imbalance in fusion and fission, and reduced mitochondrial biogenesis. *In vitro*, HL-1 atrial cardiomyocytes exposed to high glucose and palmitic acid showed excessive production of mtROS and cytosolic release of mitochondrial DNA (mtDNA), which in turn triggered cGAS-STING activation. A transwell co-culture system revealed that cardiomyocyte-derived mtDNA was engulfed by RAW 264.7 macrophages, promoting M1 polarization of macrophages and further amplifying inflammatory signaling. Importantly, pharmacological intervention with the mitochondrial antioxidant mito-TEMPO or cardiomyocyte-specific STING knockdown suppressed inflammatory responses, reversed atrial remodeling, and reduced AF susceptibility. Notably, STING overexpression sustained inflammatory pathways independently of suppressing oxidative stress, highlighting cGAS-STING signaling as a downstream effector of mitochondrial damage.

**Conclusion:** Impairment of mitochondrial quality control promotes atrial inflammation and remodeling in diabetes through mtDNA-induced cGAS-STING activation and cardiomyocyte-macrophage communication. Targeting this pathway may offer a novel strategy for AF management in metabolically compromised hearts.

## Introduction

Type 2 diabetes mellitus (T2DM), the most prevalent chronic metabolic disorder, has been established as a major predisposing factor for cardiovascular diseases including atherosclerosis, myocardial infarction, heart failure, and atrial fibrillation (AF) 1, 2. When coexisting with diabetes, AF-the most common sustained atrial arrhythmia-significantly worsens clinical outcomes by increasing stroke risk, heart failure incidence, and overall mortality 3. While the mechanisms underlying T2DM-related AF development are multifactorial, atrial structural and electrophysiological remodeling coupled with chronic inflammation have been identified as key pathological contributors, as demonstrated in our previous studies 4, 5. Given the substantial socioeconomic burden imposed by diabetes-related AF 6, elucidating its underlying mechanisms remains clinically imperative.

Growing evidence suggests that exacerbated fibrosis and inflammatory responses in diabetes-related AF may stem from disrupted mitochondrial dynamics 7 and increased monocyte/macrophage infiltration, as we previously reported 4, 5. Emerging studies reveal complex bidirectional crosstalk between macrophages and atrial myocytes in AF pathogenesis 8. Monnerat et al. demonstrated that T2DM-activated macrophages produce IL-1β, promoting ventricular arrhythmias 9. Notably, recent research has shown that upregulated cleaved N-terminal gasdermin D (NT-GSDMD) in atrial cardiomyocytes facilitates fibrosis by recruiting macrophages, creating a vicious cycle of inflammation and structural remodeling 7. These findings underscore the role of immune cell-cardiomyocyte interactions in arrhythmogenesis, but their specific implications in diabetes-related AF are not yet defined.

The cyclic GMP-AMP synthase (cGAS)-stimulator of interferon genes (STING) pathway, a pivotal regulator of innate immunity, has been demonstrated to exacerbate inflammation-induced metabolic abnormalities 10 and cardiovascular diseases 11 through its suppression. Deletion of STING specifically in cardiomyocytes markedly attenuates cardiac inflammation with concurrent improvement in mitochondrial function in diabetic cardiomyopathy 12. Cao et al. further elucidated STING's role in obesity-related AF, showing it promotes arrhythmogenesis through calcium dysregulation-induced mitochondrial damage 13. Additionally, cGAS-STING pathway inhibition reduces pro-inflammatory macrophages in atherosclerosis 14. Mechanistically, cytoplasmic double-stranded DNA (dsDNA), particularly damaged mitochondrial DNA (mtDNA) 15, acts as a damage-associated molecular pattern (DAMP) that initiates innate immunity via cGAS-STING signaling 16, amplifying cardiac inflammation and cellular dysfunction 17. Although we previously observed elevated mtDNA copy numbers in postoperative AF patients 18, whether mtDNA exacerbates T2DM-related AF through cGAS-STING activation remains unknown.

Maintenance of cardiac homeostasis relies heavily on mitochondrial quality control (MQC), which involves dynamics, mitophagy, and biogenesis. MQC dysfunction significantly contributes to diabetic cardiomyopathy by promoting mtDNA damage and leakage, thereby enhancing inflammation and oxidative stress 19, 20. Recent studies provide compelling evidence that pharmacological activation of mitophagy or stimulation of mitochondrial biogenesis pathways can effectively reduce cardiac fibrosis and improve mitochondrial function in diabetic cardiomyopathy models 21. Developing interventions to preserve MQC and prevent mtDNA leakage could provide innovative therapeutic approaches for AF induced by diabetes and other cardiovascular complications.

Our study investigates the mechanistic role of cGAS-STING activation in T2DM-related atrial remodeling and AF. Through integrated transcriptomic, *in vivo*, and* in vitro* approaches, we demonstrate that diabetes disrupts MQC, causing excessive leakage of mtDNA and subsequent elevation in phosphorylation levels of key molecules in the cGAS-STING signaling pathway in both atrial myocytes and macrophages. Importantly, we identify pathological cardiomyocyte-macrophage crosstalk as a critical driver of atrial inflammation and arrhythmogenesis. Our findings suggest that preserving MQC or targeted STING inhibition may represent promising therapeutic strategies to attenuate AF, reverse diabetes-induced atrial remodeling, and reduce AF susceptibility.

## Results

### Atrial electrophysiological and structural remodeling in T2DM mice

**Figure 1:**
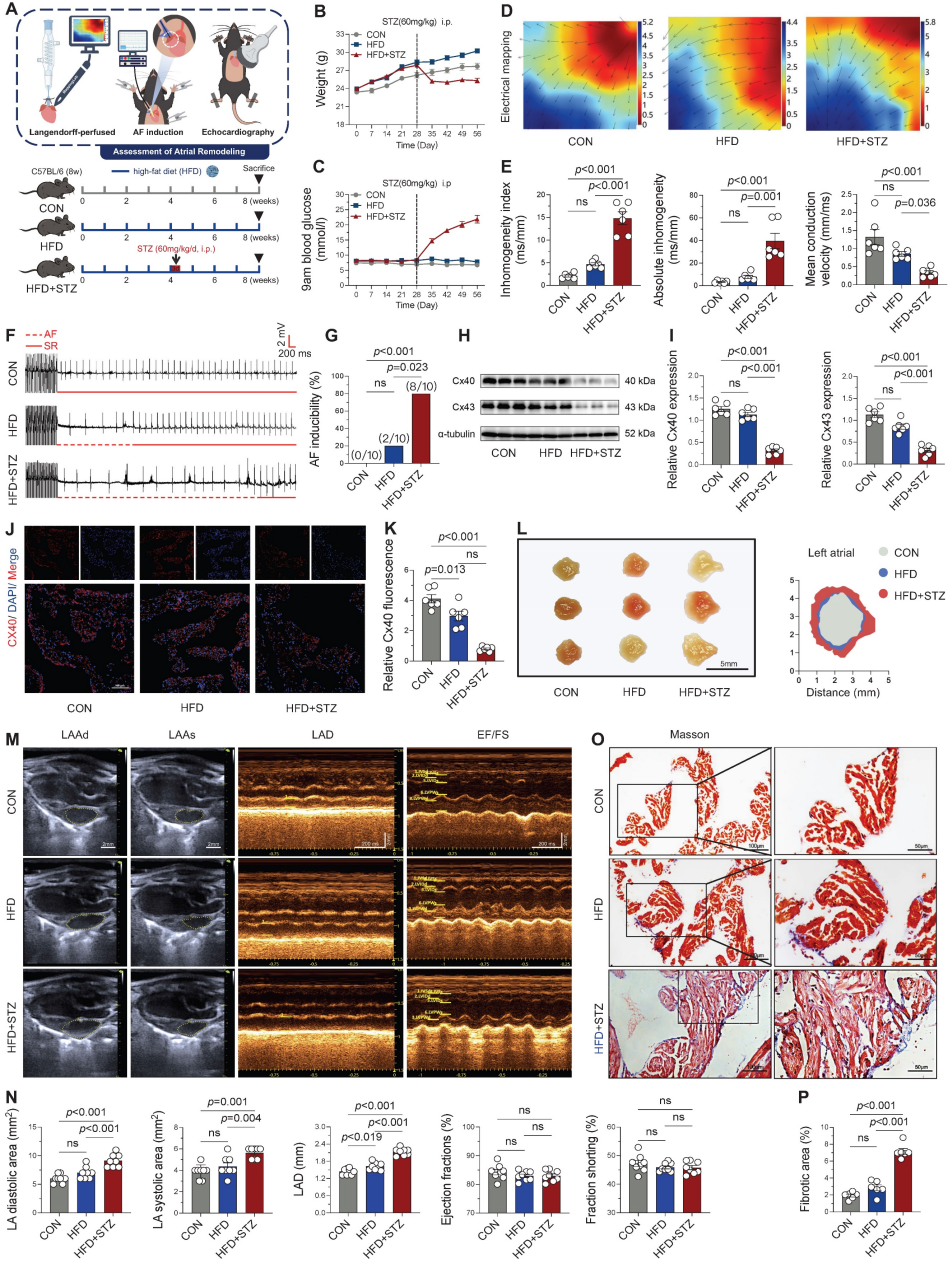
** Diabetic mice exhibited significant atrial electrical and structural remodeling, along with increased susceptibility to atrial fibrillation. (A)** Experimental design and animal group allocation. **(B)** Weekly body weight trajectories across groups (n = 12). **(C)** Weekly blood glucose profiles for all experimental groups (n = 12). **(D)** Representative left atrial conduction mapping during sinus rhythm; black arrows denote conduction direction on the epicardial surface. **(E)** Conduction characteristics of the left atrium during sinus rhythm as determined by electrophysiological mapping (n = 6). **(F)** Representative electrocardiographic tracings captured during and following atrial pacing. **(G)** AF induction success rate, defined as AF occurrence in ≥ 2 of 3 pacing attempts per mouse (n = 10). **(H-I)** Western blot analysis of atrial Cx40 and Cx43 expression levels with corresponding quantification (n = 6). **(J-K)** Immunofluorescence labeling of Cx40 (red) and nuclear DAPI (blue; scale bar = 100 μm), accompanied by quantitative analysis of Cx40 fluorescence intensity (n = 6). **(L)** Morphological depiction of left atrium (scale bar = 5mm). **(M)** Echocardiographic assessment of left atrial dimensions and ventricular function, including diastolic area (LAAd), systolic area (LAAs), diameter (LAD), ejection fraction (EF%), and fractional shortening (FS%) (scale bar = 2 mm; time scale bar = 200 ms). **(N)** Quantitative analysis of echocardiographic parameters (n = 8). (O-P) Masson's trichrome-stained left atrial sections (blue indicates fibrosis; scale bar = 100 μm) with fibrotic area quantification. The p-values were determined by one-way ANOVA followed by Tukey' s test, and error bars represent SEM. Data are expressed as mean ± SEM. AF inducibility was evaluated by Fisher's exact test.

To investigate atrial remodeling in T2DM, a model was established by combining a high-fat diet (HFD) with streptozotocin (STZ) injections (Figure 1A). T2DM mice demonstrated characteristic metabolic alterations, including significant weight loss and sustained hyperglycemia following STZ administration (Figure 1B-C), consistent with previous reports 4, validating successful model induction. Electrophysiological assessment of Langendorff-perfused hearts revealed substantial atrial remodeling in HFD + STZ group, characterized by a reduction in mean conduction velocity alongside an increase in both the inhomogeneity index and absolute conduction inhomogeneity (Figure 1D-E). Following atrial electrical stimulation, T2DM mice not only showed the highest AF incidence (80%) but also demonstrated significantly longer AF episodes compared with the other two groups (Figure 1F-G and Figure S1A). Impaired impulse conduction in atrial myocytes was further supported by diminished expression of connexin 40 in the diabetes group, as confirmed by Western blot and immunofluorescence (Figure 1H-K).

The restructuring and exacerbation of atrial fibrosis represent a key mechanism underlying the progression of AF 22. Anatomical morphology (Figure 1L) and echocardiographic analyses revealed significant atrial enlargement in the HFD + STZ group, with an enlarged atrial area and inner diameter (Figure 1M-N), while left ventricular systolic function (EF, FS) remained unaffected. Masson's trichrome staining further exhibited enhanced atrial fibrosis in diabetic mice (Figure 1O-P). These comprehensive findings demonstrate that T2DM induces profound atrial remodeling, encompassing both conduction abnormalities and increased susceptibility to atrial fibrillation, as well as structural changes, while sparing ventricular systolic function.

### Diabetic atria demonstrate significant ROS accumulation and mitochondrial quality control dysfunction

**Figure 2:**
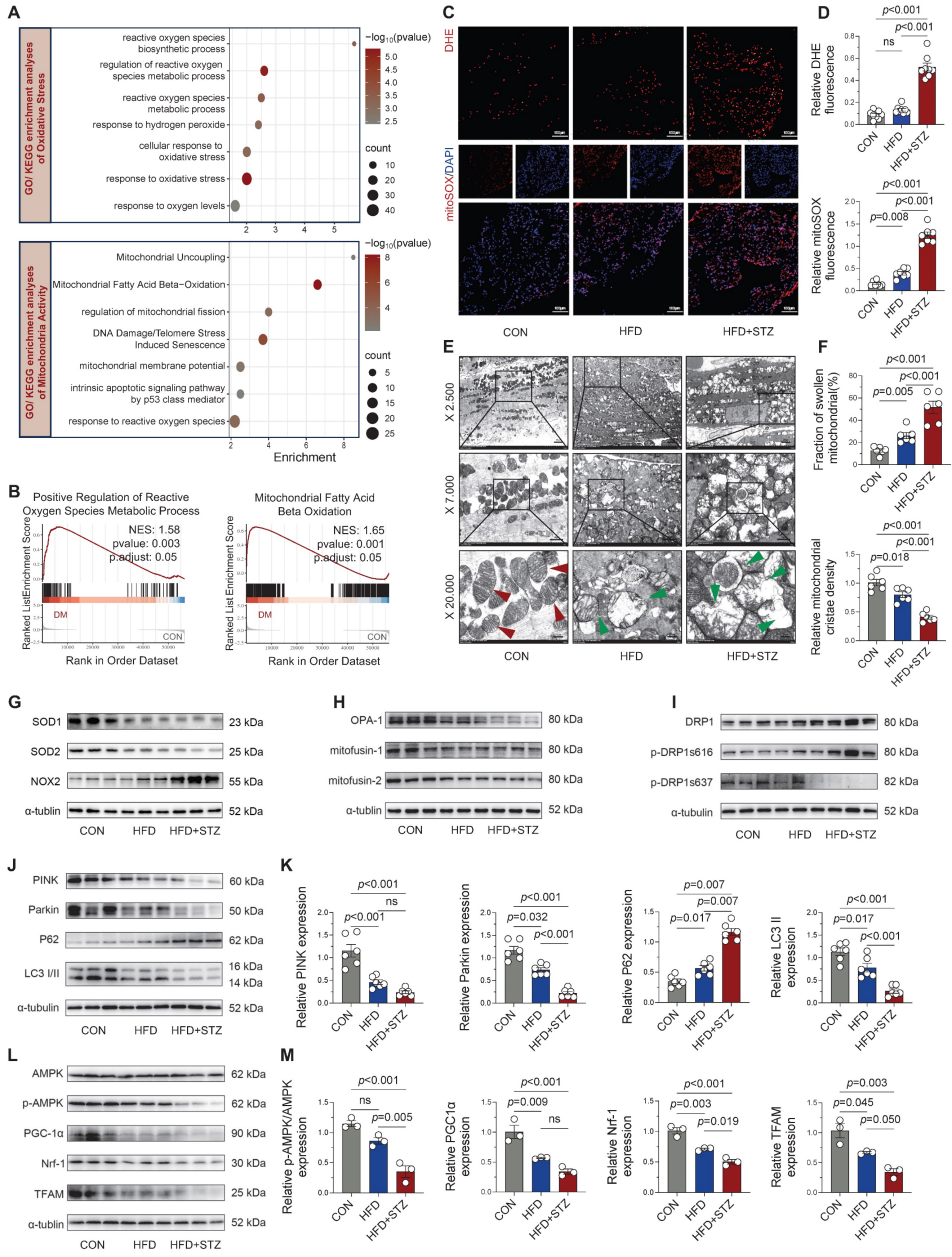
** Diabetic atria exhibited significantly increased ROS accumulation and impaired mitochondrial quality control. (A)** GO and KEGG enrichment analyses of oxidative stress and mitochondrial activity pathways. Horizontal axis shows enrichment score, with color gradient indicating p-value significance. **(B)** GSEA enrichment plots for positive regulation of ROS metabolic process and mitochondrial fatty acid β-oxidation. **(C-D)** Representative DHE and mitoSOX fluorescence micrographs of atrial sections, including quantification of fluorescence intensity (n = 6). **(E)** Transmission electron micrographs of the atria at increasing magnifications (2,500×, 7,000×, 20,000×), with red arrows indicating normal mitochondria and green arrows showing damaged mitochondria (edema, cristae disruption, vacuolization). **(F)** Quantification of mitochondrial swelling and cristae density (n = 6). **(G)** Western blot analysis of SOD1, SOD2, and NOX2. **(H-I)** Key regulators of mitochondrial dynamics: fusion proteins and fission phospho-regulation. **(J-M)** Mitophagy markers and mitochondrial biogenesis pathway components were assessed by western blot with densitometric quantification (n = 6). Statistical comparisons were performed using one-way ANOVA with Tukey's post-hoc test. Data are expressed as mean ± SEM.

Cardiac damage in diabetes is closely linked to oxidative stress and mitochondrial dysfunction, both implicated in disease progression 17. Our RNA-seq analysis of 56,482 genes identified 4,716 differentially expressed genes (3,102 upregulated and 1,614 downregulated) in diabetic atria. GO and KEGG pathway analyses revealed pronounced enrichment of oxidative stress-related pathways (including ROS metabolic/biosynthetic processes) and mitochondrial regulatory pathways (particularly fission activation) in diabetic atria (Figure 2A). GSEA further confirmed significant enrichment in ROS metabolic processes and mitochondrial fatty acid β-oxidation pathways (Figure 2B). Experimentally, we validated these findings through DHE and mitoSOX staining, which showed significantly increased fluorescence intensity in HFD + STZ atria compared to controls (Figure 2C-D), confirming elevated oxidative stress and mitochondrial ROS production.

Ultrastructural analysis by TEM revealed progressive mitochondrial damage across experimental groups, with HFD + STZ atria exhibiting the most severe abnormalities including mitochondrial swelling, cristae fractures, and severe ultrastructural disruption (Figure 2E-F). Correspondingly, the T2DM group exhibited a marked imbalance in redox-related proteins, characterized by diminished expression of SOD1 and SOD2 alongside elevated NOX2 protein levels, as shown in Figure 2G and Figure S2A.

T2DM murine atria exhibited a substantial impairment in mitochondrial quality control mechanisms. We observed significant down-regulation of fusion proteins (Figure 2H and Figure S2B) concurrent with elevated phosphorylation of DRP1 at Ser616 (Figure 2I and Figure S2C), indicating disrupted mitochondrial dynamics. Moreover, key mediators of mitophagy (Figure 2J-K) and mitochondrial biogenesis (Figure 2L-M) were significantly downregulated in HFD + STZ atria, suggesting impaired mitochondrial turnover and regeneration capacity. These findings collectively demonstrate that T2DM induces profound oxidative stress and disrupts multiple aspects of mitochondrial quality control, ultimately leading to severe mitochondrial structural and functional impairment in atrial tissue.

### cGAS-STING signaling activation and inflammatory responses in diabetic atria

**Figure 3:**
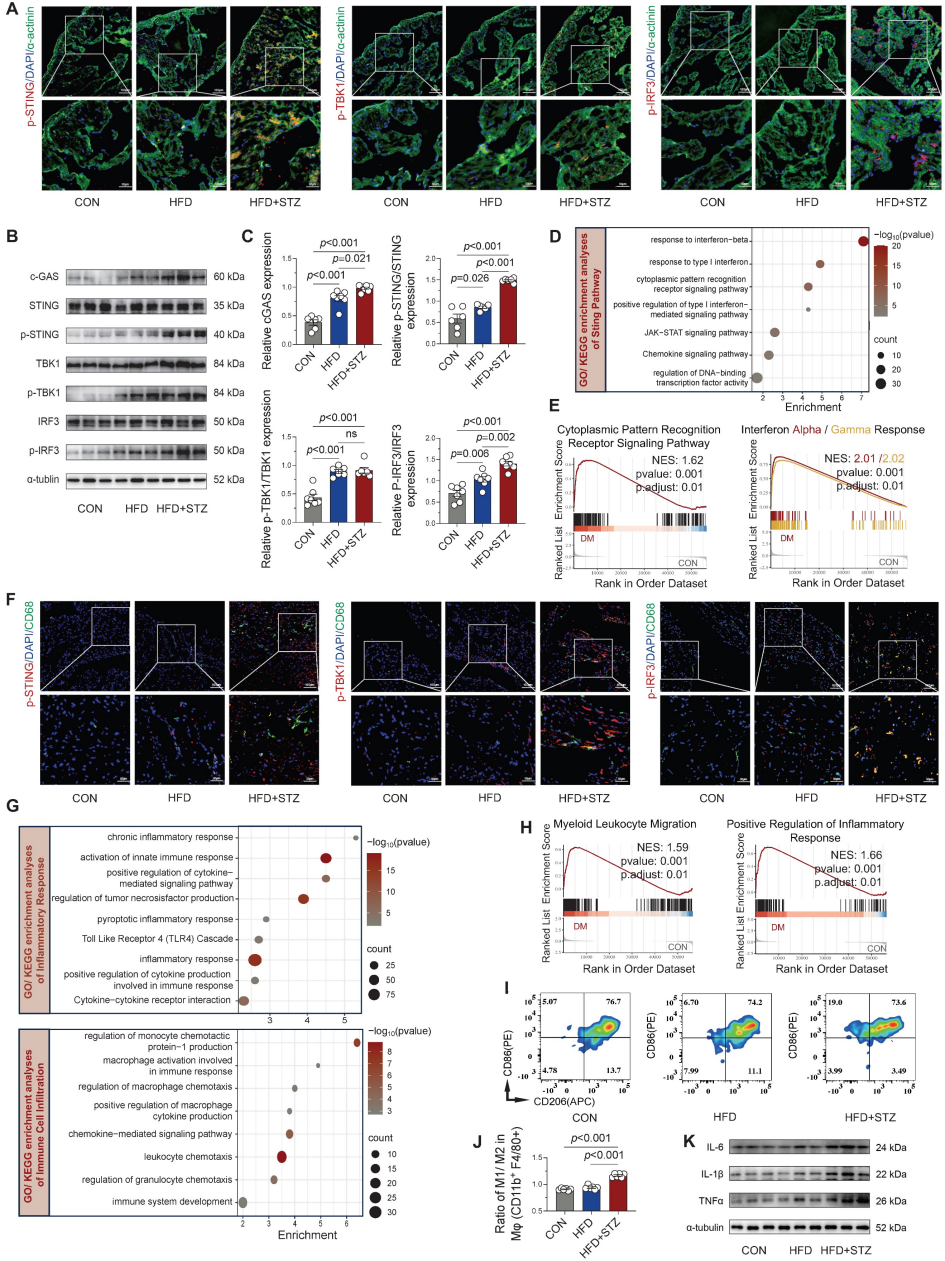
**Activation of cGAS-STING signaling and inflammatory responses in diabetic mouse atria. (A)** Immunofluorescence localization of phosphorylated STING, TBK1, and IRF3 with α-actinin (cardiomyocyte marker) and nuclear DAPI in atrial sections (scale bar = 100 μm; n = 6). **(B-C)** Western blot analysis of cGAS-STING pathway components in atrial tissue with quantitative results (n = 6). **(D)** GO/KEGG enrichment analysis of STING-related pathways. Horizontal axis shows enrichment score; color gradient indicates p-value significance. **(E)** GSEA plots for cytoplasmic pattern recognition receptor signaling and interferon-α/γ responses. **(F)** Immunofluorescence co-localization of phosphorylated STING, TBK1 and IRF3 with CD68^+^ macrophages and nuclear DAPI in atrial sections (scale bar = 100 μm). **(G)** GO/KEGG enrichment analysis of inflammatory and immune cell infiltration pathways. **(H)** GSEA plots for myeloid leukocyte migration and positive regulation of inflammatory response. **(I)** Flow cytometric profiling of immune cells in murine atrial tissue. **(J)** Quantification of M1/M2 macrophage ratio by flow cytometry (n = 6). **(K)** Western blot analysis and quantification of IL-6, IL-1β, and TNF-α protein levels. Statistical comparisons were performed using one-way ANOVA with Tukey's post-hoc test. Data are expressed as mean ± SEM.

Although mitochondrial damage-mediated STING signaling has been established as a pivotal mechanism in obesity-induced AF 13, its involvement in diabetes-associated AF pathogenesis remains unclear. Immunofluorescence analysis revealed that the phosphorylation levels of STING and its downstream key molecules were markedly elevated in atrial cardiomyocytes from diabetic mice relative to control animals (Figure 3A and Figure S3A). Western blot quantification confirmed the strongest activation of the cGAS-STING in HFD + STZ atria, showing marked phosphorylation of its key signaling components (Figure 3B-C). Transcriptomic profiling revealed substantial enrichment of interferon response and chemokine signaling pathways through GO/KEGG analysis (Figure 3D), while GSEA further confirmed upregulation of cytoplasmic pattern recognition receptor signaling and interferon-α/γ responses in diabetic atria (Figure 3E).

Furthermore, co-immunofluorescence staining revealed enhanced phosphorylation of STING/TBK1/IRF3 specifically within CD68-positive macrophages from diabetic atria (Figure 3F and Figure S3B), suggesting macrophage-specific cGAS-STING pathway activation. Functional enrichment analysis of GO/KEGG revealed chronic inflammation and immune cell infiltration and recruitment, particularly of macrophages, in diabetic atria (Figure 3G), supported by GSEA showing enhanced myeloid leukocyte migration and inflammatory responses (Figure 3H). Flow cytometric characterization of macrophage polarization states revealed a significantly increased M1/M2 ratio (CD86^+^/CD206^+^) in HFD + STZ atrial tissue versus controls (Figure 3I-J), demonstrating diabetes-induced polarization toward pro-inflammatory M1 macrophages in atrial tissue. The observed shift toward an inflammatory phenotype was supported by Western blot results, which revealed a marked increase in key inflammatory mediators within the HFD + STZ group (Figure 3K and Figure S3C). Collectively, our findings demonstrate a marked upregulation of cGAS-STING pathway in diabetic murine atria as a pivotal mechanism that drives pro-inflammatory M1 macrophage polarization and fosters a cytokine-rich environment conducive to AF pathogenesis.

### STING pathway activation with cytosolic DNA accumulation in human diabetic atria

**Figure 4:**
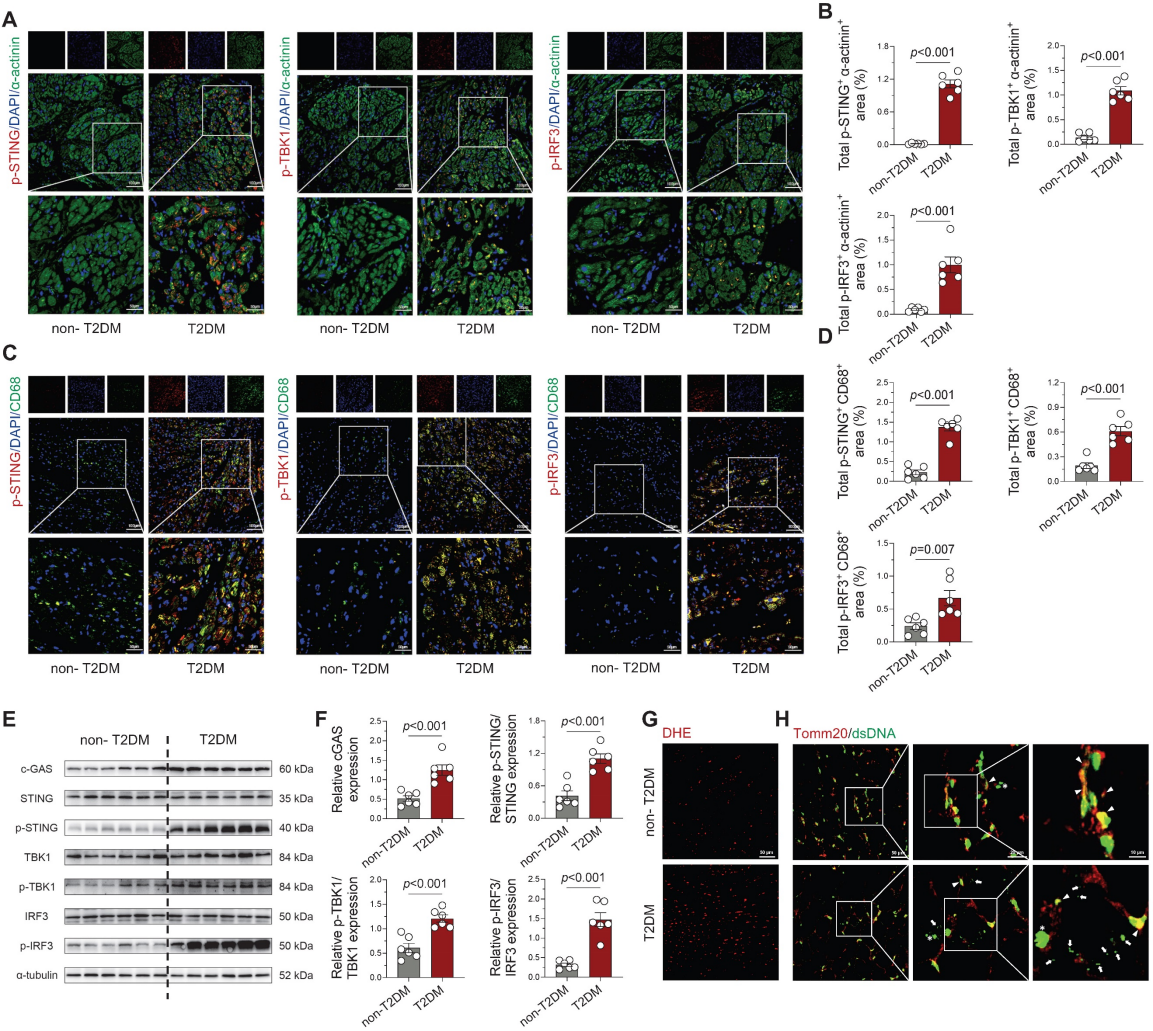
** Activation of cGAS-STING signaling pathway and cytosolic DNA accumulation in atrial tissue from T2DM patients. (A-B)** Immunofluorescence detection of phosphorylated STING, TBK1 and IRF3 with α-actinin (cardiomyocyte marker) and nuclear DAPI in human atrial specimens (scale bar = 100 μm, n = 6). **(C-D)** Macrophage-specific localization of phosphorylated STING, TBK1 and IRF3 within CD68^+^ cells counterstained with DAPI (n = 6). **(E-F)** Western blot analysis and quantification of STING, TBK1, and IRF3 phosphorylation levels in T2DM patient atria (n = 6). **(G)** DHE fluorescence imaging of oxidative stress in human atrial sections (scale bar = 50 μm). **(H)** Immunofluorescence detection of DNA (dsDNA, green) and mitochondria (Tomm20, red) demonstrates cytosolic DNA localization (scale bar = 50 μm). Asterisks indicate nuclear DNA (nDNA), arrowhead mark mitochondrial DNA (mtDNA), and arrows point to cytosolic DNA (ctDNA). The p values were determined by Student's t test, and error bars represent SEM.

Subsequently, we investigated mtDNA accumulation and cGAS-STING pathway activity in human left atrial specimens from type 2 diabetes patients (T2DM, n = 4) and non-diabetic organ donors (non-T2DM, n = 4; Table S1). Immunofluorescence analysis revealed marked phosphorylation of STING, TBK1, and IRF3 in both cardiomyocytes (Figure 4A-B) and CD68^+^ macrophages (Figure 4C-D) from diabetic patients. Western blot quantification confirmed significant elevation of these phosphorylated signaling components in diabetic atrial tissues (Figure 4E-F), paralleling our animal model observations. Concurrently, Analysis confirmed a marked increase in ROS levels in the atrial tissue of T2DM patients when comparing with non-diabetic subjects (Figure 4G). To detect cytosolic DNA not co-localizing with nuclei or mitochondria, we performed co-immuno-staining on atrial tissues using antibodies against double-stranded DNA (dsDNA) and the mitochondrial marker Tomm20. T2DM atrial tissues showed a substantial rise in scattered cytoplasmic DNA compared with non-diabetic counterparts. This suggests that severe mitochondrial and cellular damage in the diseased tissue leads to DNA leakage into the cytosol (Figure 4H).

Collectively, our analysis reveals that diabetic atrial tissues exhibit characteristic DNA damage, cytoplasmic DNA accumulation, and consequent cGAS-STING pathway activation, which likely drives macrophage polarization towards a pro-inflammatory phenotype and establishes a persistent inflammatory microenvironment conducive to AF development. Importantly, this cGAS-STING signaling activation was also observed in multiple AF models (Ang II, TAC, and AMI; Figure S4A-F), suggesting this mechanism may represent a common pathogenic pathway across diverse AF etiologies.

### STING knockdown attenuates pathway activation and inflammatory response under HG + PA stimulation

Our experimental data showed consistent cGAS-STING pathway activation in both human diabetic samples and murine T2DM tissues. To gain mechanistic insight into this pathway's contribution, HL-1 cells were subjected to a high-glucose combined with palmitic acid (HG + PA) regimen *in vitro*, a model designed to recapitulate the diabetic metabolic milieu. Optimal treatment concentrations were carefully determined by CCK-8 assays (Figure S5A). Subsequent Western blot analyses with quantitative evaluation revealed that HG + PA treatment induced overproduction of mitochondrial ROS, which subsequently impaired dysfunction in mitochondrial quality control, activated the cGAS-STING pathway, prompting inflammatory cascades activation in HL-1 cells (Figure S5B-C).

**Figure 5:**
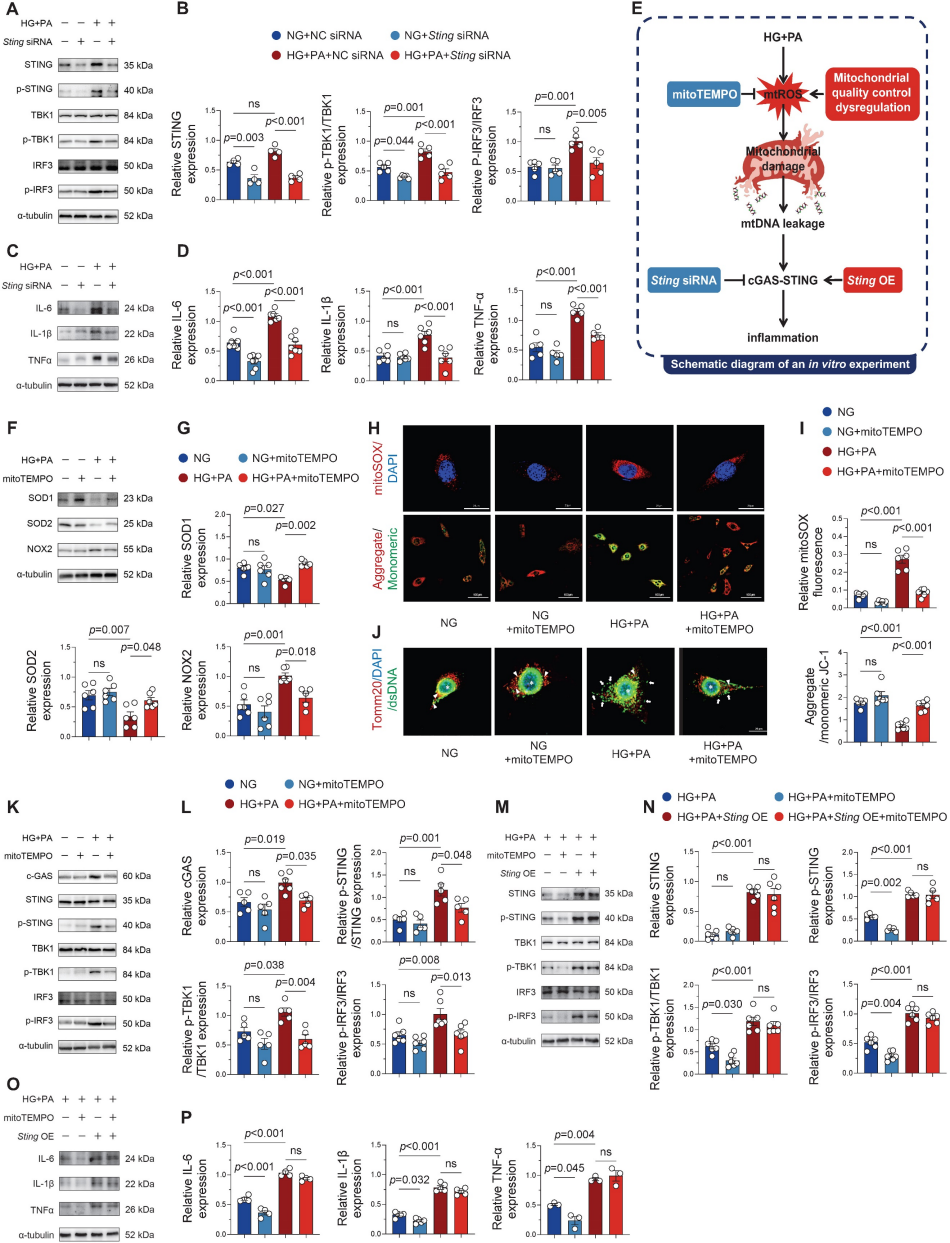
** Modulation of cGAS-STING signaling and inflammatory responses in HL-1 cells through STING overexpression and knockdown. (A-B)** Phosphorylation levels of STING, TBK1 and IRF3 in HL-1 cells assessed by Western blot with corresponding quantification (n = 6). **(C-D)** Protein expression of IL-6, IL-1β and TNF-α determined by Western blot and densitometric analysis (n = 6). **(E)** Brief schematic diagram of *in vitro* experimental procedure. **(F-G)** Western blot analysis of SOD1, SOD2 and NOX2 expression levels with quantitative evaluation (n = 6). **(H-I)** Mitochondrial superoxide production (MitoSOX Red fluorescence) and membrane potential (JC-1 aggregate/monomer ratio) visualized by fluorescence microscopy and quantified (n = 6).** (J)** Immunofluorescence staining of mitochondria (Tomm20), DNA (dsDNA), and nuclei (DAPI) in HL-1 cells. Asterisks indicate nuclear DNA (nDNA), arrowhead mark mitochondrial DNA (mtDNA), and arrows point to cytosolic DNA (ctDNA, bar = 20 μm). **(K-L)** Phosphorylation levels of STING, TBK1 and IRF3 in HL-1 cells assessed by Western blot with corresponding quantification (n = 6). **(M-N)** Representative western blot images and quantitative analysis of p-STING, p-TBK1, p-IRF3 in HL-1 cells (n = 6). **(O-P)** Protein expression of IL-6, IL-1β and TNF-α determined by Western blot and densitometric analysis (n = 6). Statistical comparisons were performed using one-way ANOVA with Tukey's post-hoc test. Data are expressed as mean ± SEM.

To ascertain the functional contribution of this pathway, we performed STING-specific siRNA knockdown in HG + PA-treated HL-1 cells. Strikingly, genetic suppression of STING effectively suppressed downstream signaling within the cGAS-STING axis (Figure 5A-B) and concomitantly lowered the expression of pro-inflammatory cytokines (Figure 5C-D). Collectively, our results demonstrate the critical involvement of the cGAS-STING pathway in propagating the inflammatory response in HG + PA-exposed cardiomyocytes.

### STING overexpression circumvented mitochondrial antioxidant defense to maintain inflammatory response under HG + PA stimulation

Mito-TEMPO mediates its protective effects against AF susceptibility and duration primarily through suppression of mitochondrial ROS overproduction and reduction of cardiomyocyte apoptosis 7. To elucidate the underlying molecular mechanisms, we treated HL-1 cardiomyocytes with HG + PA in the presence or absence of mito-TEMPO, systematically assessing oxidative stress markers, mitochondrial integrity, cGAS-STING pathway activation, and inflammatory responses (Figure 5E). Western blot analyses showed that HG + PA challenge induced profound oxidative stress, as evidenced by suppressed antioxidant enzymes and elevated pro-oxidant NOX2 expression (Figure 5F-G)-effects that were effectively reversed by mito-TEMPO treatment. Consistent with these findings, MitoSOX fluorescence assays demonstrated that HG + PA-induced mtROS overproduction was substantially attenuated by mito-TEMPO (Figure 5H-I). Further mechanistic investigations revealed that HG + PA treatment compromised mitochondrial membrane potential (as indicated by a reduced JC-1 aggregate/monomer ratio) and promoted cytoplasmic dsDNA leakage (Figure 5J), both pathological manifestations of mitochondrial dysfunction that were significantly ameliorated by mito-TEMPO administration. Notably, mito-TEMPO treatment also effectively suppressed HG + PA-induced activation of the cGAS-STING inflammatory pathway and downstream proinflammatory factors (Figure 5K-L). Collectively, these findings demonstrate that HG + PA treatment disrupts mitochondrial homeostasis through excessive mtROS generation, thereby triggering mtDNA fragment release and consequent cGAS-STING signaling engagement. Importantly, pharmacological intervention with mito-TEMPO effectively preserves mitochondrial integrity and suppresses cGAS-STING pathway activation.

To further elucidate STING's role in maintaining inflammation, we overexpressed STING in HG + PA + mito-TEMPO-treated HL-1 cells. Strikingly, STING overexpression completely restored cGAS-STING pathway activity (Figure 5M-N) and pro-inflammatory cytokine production (Figure 5O-P) despite mito-TEMPO co-treatment. This persistent inflammatory response under combined treatment conditions suggests that STING activation operates downstream of mtROS-mediated signaling and can independently sustain inflammatory cascades even when oxidative stress is pharmacologically controlled. These *in vitro* findings strongly suggest that effective treatment of diabetes-associated atrial inflammation may require dual targeting of both mitochondrial oxidative stress and the cGAS-STING signaling pathway.

### Macrophage cGAS-STING pathway activation by mitochondrial DNA (mtDNA) from injured cardiomyocytes

**Figure 6:**
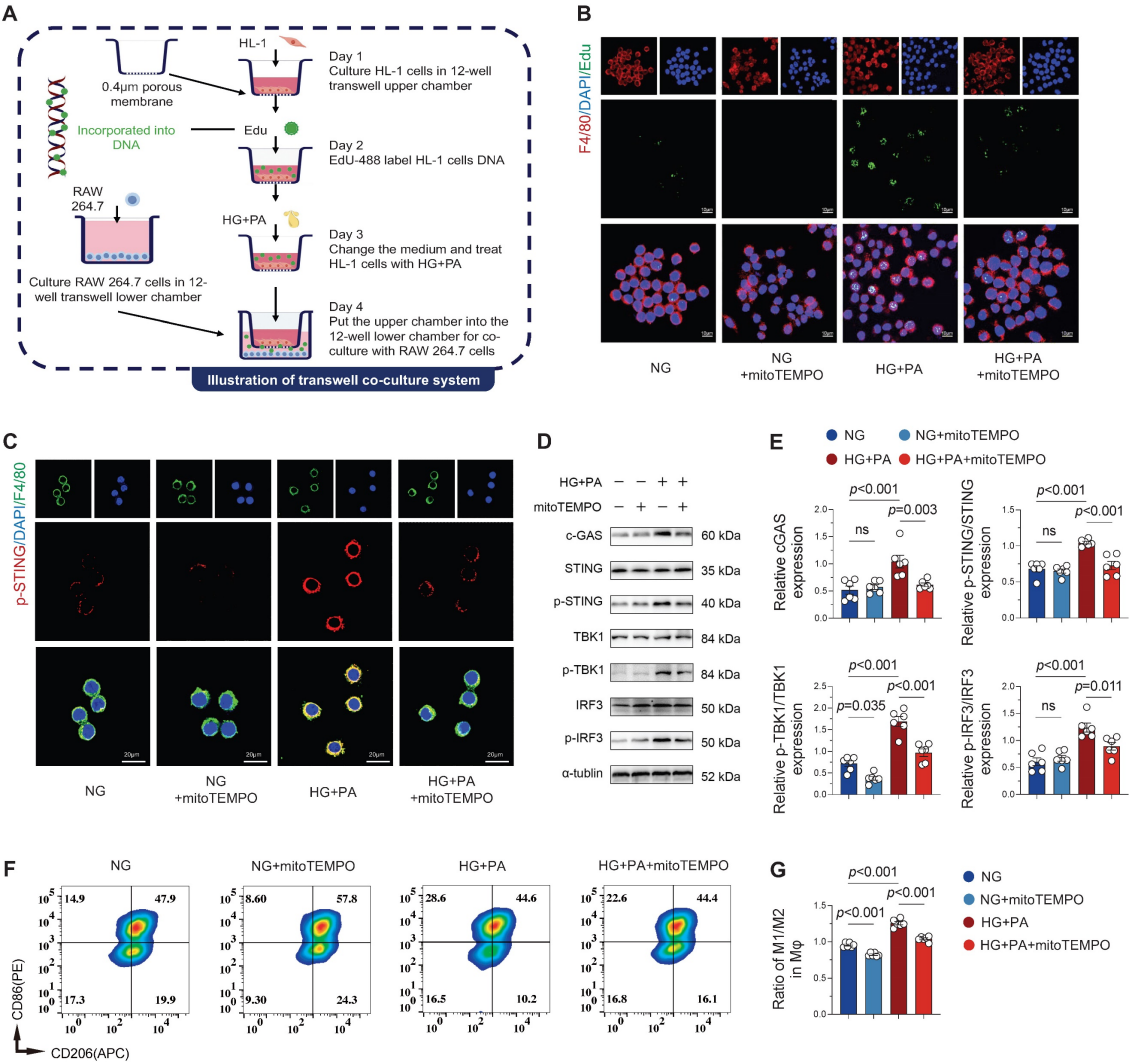
**Atrial myocyte-derived DNA promotes macrophage M1 polarization via STING signaling. (A)** Schematic of transwell co-culture system design (0.4 μm pore size membrane separating HL-1 cardiomyocytes in upper chamber from macrophages in lower chamber) and experimental timeline. **(B)** Immunofluorescence detection of EdU-labeled DNA (from HG + PA-treated HL-1 cells) in F4/80^+^ macrophages following co-culture, demonstrating intercellular DNA transfer (scale bar = 10 μm). **(C)** Immunofluorescence staining for p-STING (red), F4/80 (green) and DAPI (blue) in RAW 264.7 cells (scale bar = 20 μm). **(D-E)** Western blot analysis and quantification of cGAS-STING pathway activation in co-cultured macrophages (n = 6). **(F)** Representative flow cytometry plots of macrophage polarization markers. **(G)** Quantitative analysis of M1/M2 macrophage ratio by flow cytometry (n = 6). Statistical comparisons were performed using one-way ANOVA with Tukey's post-hoc test. Data are expressed as mean ± SEM.

Building upon previous findings demonstrating AF-induced macrophage recruitment and pro-inflammatory cytokine secretion that exacerbates local inflammation 5, 23, we investigated whether cardiomyocyte-derived mitochondrial DNA (mtDNA) serves as a pro-inflammatory stimulus for macrophages. Utilizing a transwell co-culture system of atrial cardiomyocytes (HL-1) and macrophages (RAW 264.7), we first confirmed the transfer of DNA labeled by Edu (green fluorescence) from HL-1 cells to macrophage cytosol (Figure 6A), demonstrating active phagocytosis of cardiomyocyte-derived DNA by macrophages. Immunofluorescence quantification showed significantly increased EdU signal in macrophages co-cultured with HG + PA-treated HL-1 cells, while mito-TEMPO treatment substantially reduced this signal (Figure 6B), demonstrating that mitigating mitochondrial damage in cardiomyocytes decreases extracellular DNA release. Co-cultured macrophages exhibited substantially suppressed cGAS-STING pathway activation when exposed to mito-TEMPO-treated HL-1 cells, relative to those co-cultured with HG + PA-stimulated cardiomyocytes (Figure 6C-E). Flow cytometric assessment of macrophage polarization revealed the greatest M1/M2 ratio (CD86^+^/CD206^+^) in RAW 264.7 cells when exposed to HG + PA-treated HL-1 cells. Despite partial normalization with mito-TEMPO treatment, this ratio remained heightened in comparison to the control (NG) and NG + mito-TEMPO groups (Figure 6F-G). These* in vitro* findings collectively indicate that damaged atrial myocytes release extracellular DNA that drives pro-inflammatory M1 macrophage polarization and subsequent inflammatory activation.

### STING knockdown suppresses atrial remodeling and ameliorates AF susceptibility in the diabetic murine model

**Figure 7:**
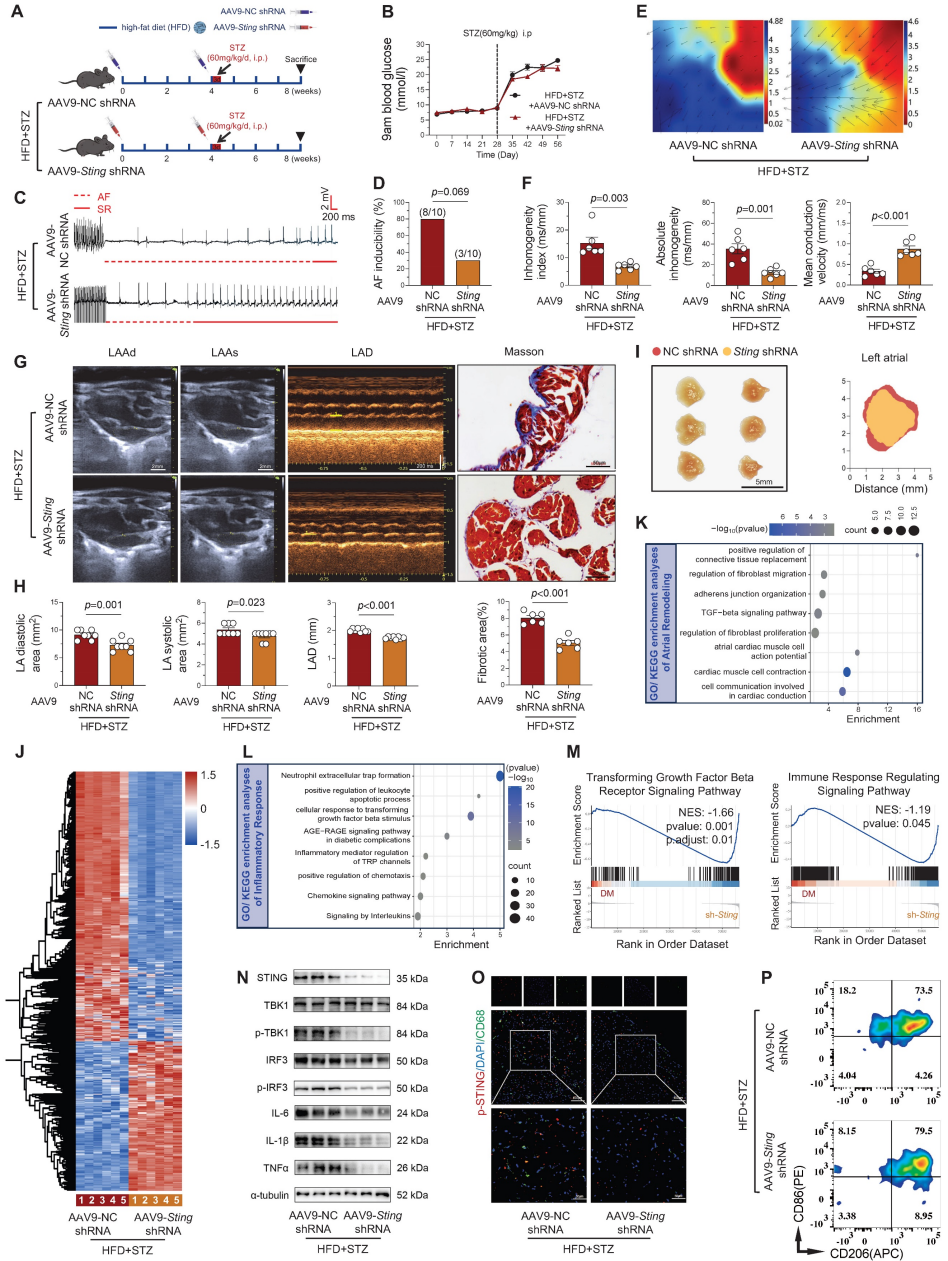
** STING knockdown attenuates atrial remodeling and inflammation in diabetic mice. (A)** Experimental design schematic and animal group allocation. **(B)** Weekly blood glucose monitoring across groups (n = 12). **(C)** Representative ECG traces showing AF induction by programmed electrical stimulation, with transition from AF (dashed red line) to sinus rhythm (solid red line). **(D)** AF incidence rates across experimental groups (n = 10). **(E-F)** Atrial electrophysiological mapping during sinus rhythm with EMapScope 5.0 software (n = 6) **(G)** Echocardiographic measurements (LAAd, LAAs, LAD) and Masson's trichrome-stained atrial sections (ultrasound scale = 2 mm; histology scale = 50 μm). **(H)** Quantitative echocardiographic (n = 8) and fibrotic area analysis (n = 6). **(I)** Morphological depiction of the left atrium (scale bar = 5 mm). **(J)** Heatmap of atrial RNA-seq differentially expressed genes (DEGs, 792 upregulated, 1,429 downregulated). **(K-L)** GO/KEGG enrichment analysis of atrial remodeling and inflammatory pathways. **(M)** GSEA plots for TGF-β receptor signaling and immune response regulation pathways. **(N)** Western blot analysis of cGAS-STING pathway components in atrial tissue with quantitative results (n = 6). **(O)** Macrophage-specific STING phosphorylation (p-STING) in CD68^+^ cells counterstained with nuclear DAPI in atrial sections (scale bar = 50 μm). **(P)** Representative images of flow cytometry analysis of mouse atria. The p values were determined by Student's t test, and error bars represent SEM. AF inducibility was evaluated by Fisher's exact test.

Building upon our* in vitro* findings demonstrating that STING knockdown mitigates HG + PA-induced pro-inflammatory factor release in atrial myocytes, we next investigated its therapeutic potential *in vivo* using AAV9-mediated myocardial-specific STING knockdown in T2DM mice (Figure 7A). While cardiac STING knockdown did not affect hyperglycemia in diabetic mice (Figure 7B), it significantly reduced AF susceptibility, including reduced AF inducibility and shortened AF duration (Figure 7C-D and Figure S7A).

To delineate the pathological mechanisms, we validated changes in atrial electrical remodeling and structural enlargement. Langendorff perfusion experiments demonstrated that STING knockdown (HFD + STZ + *shSting* group) significantly decreased absolute atrial conduction inhomogeneity and the inhomogeneity index, while also increasing mean conduction velocity (Figure 7E-F). Ultrasound measurements revealed that targeting STING downregulation reduced diabetes-induced atrial enlargement, manifested by decreased atrial area and diameter (Figure 7G-H and Figure S7B), accompanied by alterations in atrial morphology (Figure 7I). Histological analysis confirmed diminished atrial fibrosis in STING knockdown mice (Figure G-H). Transcriptomic profiling of atrial tissue indicated that STING knockdown attenuated profibrotic pathways, particularly through suppression of TGF-β signaling and inhibition of fibroblast proliferation in diabetic mice (Figure 7K). These comprehensive findings establish that cardiac-specific STING inhibition not only ameliorates diabetes-induced electro-physiological remodeling but also mitigates structural changes including atrial dilation and fibrosis, highlighting STING as a promising therapeutic target for diabetic-related AF.

### STING knockdown suppresses atrial inflammation and macrophage M1 polarization in diabetic mice

To elucidate the anti-inflammatory mechanisms of STING knockdown, we performed comprehensive transcriptomic and functional analyses. Atrial tissue RNA sequencing of atrial tissue identified 2,221 differentially expressed genes (792 upregulated, 1,429 downregulated) upon STING knockdown (Figure 7J). GO/KEGG enrichment analyses involved the AGE-RAGE signaling pathway in diabetic complications and the chemokine signaling pathway (Figure 7L). GSEA further confirmed suppression of key pro-fibrotic and inflammatory pathways, notably transforming growth factor beta receptor signaling and immune response regulation (Figure 7M).

Western blot analysis confirmed substantial downregulation of cGAS-STING pathway components and inflammatory mediators in HFD + STZ + *shSting* mice compared to HFD + STZ + shNC controls (Figure 7N and Figure S7C). Additionally, immunofluorescence staining revealed significantly reduced STING phosphorylation in CD68^+^ macrophages from STING knockdown atria (Figure 7O and Figure S7D). Flow cytometry revealed a significant reduction in M1 macrophage proportion, accompanied by decreased pro-inflammatory cytokine levels in STING-deficient samples (Figure 7P and Figure S7E). These findings collectively demonstrate that cardiomyocyte-specific STING knockdown effectively mitigates diabetes-associated atrial inflammation by suppressing macrophage pro-inflammatory polarization through modulation of multiple signaling pathways.

## Discussion

Our findings establish disrupted MQC and cGAS-STING-driven inflammation as key drivers of progressive atrial remodeling and enhanced AF vulnerability in the diabetic context. We identified consistent upregulation of cGAS-STING signaling components both in diabetic atrial tissues and across multiple AF models (AngII infusion, TAC, and AMI), suggesting its fundamental involvement in arrhythmogenesis. Using an* in vitro* transwell co-culture system, we elucidated a pathogenic interaction wherein atrial myocytes release excessive dsDNA that promotes macrophage M1 polarization, jointly contributing to proinflammatory responses. Both genetic STING knockdown and pharmacological intervention with mito-TEMPO effectively attenuated inflammatory responses in HG + PA-injured atrial myocytes by suppressing cGAS-STING activation, while STING overexpression maintained inflame-matory responses despite antioxidant treatment, demonstrating pathway dominance. Most significantly, cardiac-specific STING knockdown via AAV in diabetic mice substantially improved atrial electrophysiological and structural remodeling while suppressing M1 polarization of macrophages and inflammation, establishing STING inhibition as a promising therapeutic strategy.

T2DM promotes atrial arrhythmogenesis through interconnected mechanisms, with mitochondrial dysfunction and inflammation as central mediators. ROS-mediated mitochondrial damage triggers pro-inflammatory cytokine release 24, 25, coupled with macrophage-driven inflammatory responses significantly contribute to AF development 5. As cellular powerhouses, mitochondria are crucial for maintaining cardiac electrophysiological stability 26-28. Research from our group and others has documented that mitochondrial oxidative stress in diabetic atria disrupts the balance of mitochondrial dynamics, leading to increased fission and suppressed fusion that markedly worsens atrial remodeling 4. While mitochondrial dynamics in diabetes-related AF are increasingly understood, the roles of mitophagy and biogenesis-particularly in damaged mitochondrial clearance-remain unclear. Tong et al. discovered that impaired mitophagy exacerbates mitochondrial dysfunction, lipid accumulation, and worsens diabetic cardiomyopathy 29. Moreover, previous research establishes mitochondrial impairment in cardiac metabolic disorders as a key trigger for cGAS-STING pathway activation 30. Recent research indicates that administering Pioglitazone improves mitochondrial biogenesis through PPAR-γ/PGC-1α activation, effectively reducing AF burden in diabetic mice 31. In this study, a notable increase in mitochondrial oxidative damage and Drp1-dependent fission was observed in the atria of diabetic mice, alongside a significant decrease in mitochondrial fusion, mitophagy, and biogenesis. Furthermore, mitochondrial dysfunction may directly promote arrhythmogenesis by disrupting electrical conduction. Emerging evidence indicates that such dysfunction can slow conduction velocity through mechanisms including reduced sodium current and impaired intercellular coupling 32. These data implicate altered mitochondrial dynamics and mitophagy in the pathogenesis of atrial remodeling, highlighting the maintenance of mitochondrial homeostasis as a potentially crucial intervention for diabetic cardiovascular complications, especially arrhythmias, which warrants further exploration.

Mitochondrial dysfunction in metabolic heart diseases leads to the release of mtDNA fragments, which is strongly associated with disease severity and key pathological features including inflammation, ROS burden and insulin resistance 33. These cytosolic mtDNA leaks activate cGAS-STING pathway, triggering innate immunity 34, 35. Our study reveals significant cGAS-STING pathway activation in diabetic atria. This pathway has been implicated in aortic dissection 36, diabetic cardiomyopathy 37, Doxorubicin-Induced cardiotoxicity 38 and obesity-induced arrhythmias 13. For instance, treatment of vascular endothelial cells with PA induces mitochondrial damage, prompting the release of mtDNA, which activates cGAS-STING and subsequently triggers interferon production 39. Wang et al. provided key evidence that STING pathway blockade attenuates hypertrophic responses in primary cardiomyocytes cultured from pathological myocardial tissue 40. While the cGAS-STING pathway's role in diabetic AF remains unclear, studies show that STING knockout or inhibition reduces diabetes-related cardiac inflammation by suppressing NLRP3 inflammasome activation 41. Furthermore, Cao et al. found that STING knockdown partially improved reduced ICa,L density and calcium transient amplitude in obese rats, consequently attenuating atrial remodeling and AF susceptibility 13. Extending previous observations of cGAS-STING activation in diabetic inflammation, our findings position mitochondrial damage as a critical trigger of this pathway in driving atrial inflammation under diabetic conditions.

Bioinformatic analyses in this study revealed pathways associated with oxidative stress, mitochondrial dysfunction, STING signaling activation, inflammatory responses, and immune cell infiltration, as indicated by GO and KEGG enrichment. Furthermore, GSEA showed upregulation of cytoplasmic pattern recognition receptor signaling and interferon responses. Consistent with findings from animal and cellular experiments, it was established that impaired MQC and cGAS-STING pathway activation critically contribute to atrial structural remodeling and arrhythmogenesis in diabetic conditions. Nevertheless, despite this evidence, current knowledge regarding potential pharmacological interventions targeting MQC defects and cGAS-STING activation in diabetic atria remains substantially limited.

Macrophages play a central role in AF pathogenesis through innate immune functions. Numerous studies demonstrate increased macrophage infiltration in AF tissues compared to other cell types, where they promote inflammation and fibrosis by disrupting cardiomyocyte electrical coupling 42. Recent research by Hulsmans et al. revealed that macrophage-derived SPP1 activates cardiac fibroblasts, driving pro-fibrotic and inflammatory responses 23. These findings underscore the critical role of macrophage-cardiomyocyte or fibroblast crosstalk in promoting structural remodeling through impairment of atrial electrical conduction. In this study, atrial enlargement was observed to be partly attributable to impaired ventricular diastolic function, which appears mechanistically connected to the metabolic disturbances and interstitial fibrosis. Notably, both clinical and experimental studies show atrial cardiomyocytes recruit macrophages via IL-1β secretion 7, while our *in vitro* co-culture system reveals macrophages respond to atrial myocyte-derived mtDNA through cGAS-STING activation, amplifying inflammatory cytokine production. This mechanism parallels observations in ischemic injury, where nuclear DNA leakage activates macrophage cGAS-STING pathways to promote pro-inflammatory M1 polarization, while STING inhibition favors reparative M2 phenotypes 43. Furthermore, we observed limited alterations in neutrophil infiltration and N1 polarization within the diabetic atria, suggesting that macrophage infiltration and M1 polarization may constitute the predominant inflammatory response under these conditions. Similarly, study by Luo et al. in aortic dissection models show macrophage processing of smooth muscle cell DNA fragments into cGAMP upregulates MMP-9-mediated extracellular matrix degradation 36. Despite these advances, the precise mechanisms governing cardiomyocyte-macrophage interactions and the pathogenetic contributions of cGAS-STING signaling in diabetes-associated AF remain incompletely characterized.

Our results show significant cGAS-STING activation in AF models and HG + PA-treated cardiomyocytes, driven by impaired mitochondrial quality control. These findings align with reports in myocarditis 44, hypertrophic cardiomyopathy 45, and obesity-associated AF. Importantly, genetic *Sting* knockdown attenuates inflammatory, reverses atrial dilatation and fibrotic remodeling, reduces M1 macrophage polarization, and ultimately decreases AF susceptibility in diabetic mice. Pharmacological intervention with mito-TEMPO similarly mitigates mitochondrial dysfunction and subsequent cGAS-STING activation, effects that are counteracted by STING overexpression. Our results identify cGAS-STING signaling as a key driver of AF in diabetic contexts and reveal its translational relevance for developing anti-arrhythmic strategies. Moreover, in transwell co-culture systems, mito-TEMPO-treated atrial myocytes substantially reduce M1 macrophage polarization and cGAS-STING activation in underlying macrophages, identifying a novel dsDNA-mediated cardiomyocyte-macrophage crosstalk mechanism in metabolic arrhythmogenesis. Notably, previous studies have shown that regulating crosstalk between macrophages and cardiomyocytes has been implicated in post-MI cardiac repair 46 and conduction system homeostasis 47. To our knowledge, this work identifies a previously unrecognized pathway connecting diabetes to AF susceptibility, thereby advancing our comprehension of metabolic influences on cardiac rhythm.

There are several limitations in our study. Firstly, although we demonstrated that mitochondrial protection significantly attenuates cGAS-STING pathway activation, the direct evidence linking mitochondrial dsDNA release to cGAS-STING signaling initiation remains to be conclusively established. Our investigation into the interaction between cardiomyocytes and macrophages is confined to *in vitro* experiments, with a lack of *in vivo* studies to further explore and validate. Secondly, while our work focused on macrophage polarization, it did not fully explore the role of other immune cells, including neutrophil subsets, in AF pathogenesis. The potential interplay between different immune cell populations in the diabetic atrium remains an important area for future investigation. Additionally, our findings establish cGAS-STING signaling as a key driver of AF in diabetic contexts. However, translating this strategy into clinical practice requires careful consideration of challenges such as achieving tissue-specific targeting and ensuring long-term immune safety 48.

## Conclusion

In summary, our study showcases notable activation of the cGAS-STING signaling pathway in diabetic atrial tissue. This pathway activation appears primarily driven by mitochondrial DNA release from damaged cardiomyocytes, which initiates a pathogenic cardiomyocyte-macrophage crosstalk that plays a central role in diabetes-induced atrial remodeling. These findings suggest that targeted modulation of the cGAS-STING pathway may represent a novel therapeutic strategy for diabetes-associated atrial fibrillation.

## Methods and Materials

### Human atrial samples

Left atrial specimens were obtained from male patients undergoing surgical procedures at the General Hospital of the Northern Theater Command. Samples from the T2DM group were obtained from patients diagnosed with type 2 diabetes, while samples from the non-T2DM group were from heart donors. Informed consent was obtained from all patients or their family members. Patients with severe hepatic or renal dysfunction, immunodeficiency diseases, infectious diseases, or cancer were excluded. This study was conducted following approval from the Ethics Committee of the General Hospital of the Northern Theater Command (Y (2023) 094, 17 May 2023) and in accordance with the Declaration of Helsinki.

### Animal and treatment

Eight-week-old male wild-type C57BL/6J mice from the same batch were supplied by Beijing HFK Bioscience Co., Ltd. All animal experiments were approved by the Ethics Committee of the General Hospital of the Northern Theater Command (Approval No. 2023-47; December 28, 2023) prior to initiation. Following acclimation under controlled conditions (25 ± 3 °C, 12 h light/dark cycle) with free access to food and water, mice were subjected to a standardized protocol for establishing a type 2 diabetes mellitus (T2DM) model 4. Diabetic status was confirmed in mice with fasting blood glucose levels above 16.7 mmol/L. Serial measurements of body mass and blood glucose were performed at scheduled time points during the experimental protocol. Additionally, the AngII infusion, TAC surgery, and AMI mouse models used in this study were generated according to established protocols 49-51.

### Bulk RNA sequencing

Following euthanasia, atrial tissue samples were promptly collected and immersed in RNA protect reagent. Total RNA was then extracted using the RNeasy miRNA Kit according to the manufacturer's protocol 49. Subsequently, RNA sequencing libraries were constructed and sequenced as previously described 52. Each experimental group included six biologically independent replicates for sequencing.

### RNA-seq data analysis

FastQC (version 0.11.9) was utilized for the quality control evaluation of the raw sequencing reads from the mouse atrial transcriptomic data. Transcript abundance was determined via featureCounts (v2.0.3), with all subsequent analyses performed in R (RStudio 2023.06.0 Build 421) using an adapted analytical approach 53. DEGs analysis was performed utilizing the "limma" package, with criteria established at |Log2FC| > 0.263 and adjusted p-value < 0.05. A heatmap visualization was produced utilizing TBtools-II software 54. Functional annotation of the mouse atrial transcriptome was performed via comprehensive enrichment analyses for GO terms and KEGG pathways, utilizing the DAVID bioinformatics platform (version 3.5.20240101) 55. GSEA was further carried out on the GSEA platform (version 4.3.3).

### Adeno-associated virus (AAV) transfection* in vivo*

For *in vivo* assessment of *Sting* function, an AAV9 vector carrying cardiac troponin T (cTnT) promoter-driven Sting-specific shRNA (AAV-*Sting* shRNA) or scrambled control shRNA (AAV-NC shRNA) was designed and packaged by Hanbio Biotechnology Co., Ltd. (Shanghai, China). To enable cardiomyocyte-restricted *Sting* knockdown, 100 μL of AAV9 (viral titer: 2 × 10¹² vg/mL) was delivered via tail vein injection. The virus was administered twice to ensure efficient *Sting* knockdown: once the day before starting a high-fat diet (HFD) and again the day before the initial STZ injection (Figure 7A). Western blot analysis performed four weeks after the second viral injection confirmed specificity and efficient knockdown of Sting in cardiomyocytes.

### Echocardiography measurements

Transthoracic echocardiography was carried out on the mice using a high-resolution ultrasound imaging system (D700, Vinno Technology) equipped with a dedicated small-animal cardiac phased-array transducer. Mice were anesthetized via inhalation of 2% isoflurane to maintain stable sedation throughout the examination.

Cardiac function was assessed with a focus on left atrial and ventricular performance, using specific metrics detailed in our previous study 56. Data acquisition spanned a minimum of six consecutive cardiac cycles to enhance accuracy and reproducibility. EF and FS values were automatically computed by built-in software algorithms. All analyses were conducted in a blinded fashion to minimize potential bias.

### Electrocardiogram (ECG) recording and AF induction

Mice were placed under general anesthesia via an intraperitoneal injection of pentobarbital sodium (50 mg/kg) and connected to a small animal ventilator for respiratory support. Continuous surface electrocardiographic (ECG) monitoring was acquired with an 8-channel PowerLab system (AD Instruments) 57. AF was elicited by advancing an electrode catheter (Transonic Scisense) through the right external jugular vein into the right atrium. The stimulation parameters comprised an amplitude of 5 V, a cycle length of 40 ms, a pulse width of 5 ms, and 10 bursts. AF episodes were identified by sustained (> 1 s) irregular atrial rhythms with replacement of distinct P-waves by fibrillatory waves on ECG. Successful AF induction was confirmed when ≥ 2 out of 3 pacing attempts elicited AF episodes.

### Langendorff-perfused hearts

Following euthanasia, the mice were subjected to thoracotomy, and their hearts were perfused with oxygenated Tyrode's solution using a Langendorff-perfusion system. Tyrode's solution was formulated and prepared according to a previously established protocol 58. The high-density 64-channel microelectrode array (MappingLab Ltd.) was precisely placed perpendicular to the surface of the left atrial region and recorded signals simultaneously. Data mapping was carried out with EMapScope software, and subsequent data analysis was conducted to derive parameters reflective of atrial electrical conduction, as previously described 57.

### Transmission electron microscopy (TEM) sample preparation and analysis

The freshly isolated left atrial tissues were promptly immersed in an ice-cold glutaraldehyde solution (G1102, Servicebio Technology), prepared in 0.1 M phosphate buffer (pH 7.4), and fixed at 4 °C for 3 h. Following primary fixation, the specimens were processed through sequential gradient ethanol dehydration, resin embedding, and ultrathin sectioning. Ultrastructural analysis was conducted on a transmission electron microscope (OLYMPUS, Japan). For each tissue section, four fields were randomly selected and systematically examined across a range of magnifications (2500× to 20,000×) to allow detailed assessment of mitochondrial morphology. Quantitative assessment of mitochondrial abnormalities, including cristae disruption and matrix swelling, was performed by morphological analysis using ImageJ software. The proportion of swollen mitochondria and the corresponding cristae density were quantified in accordance with established methodologies 27, 59.

### Masson's trichrome staining

The atrial tissue specimens were initially fixed with 4% paraformaldehyde (C104188, Aladdin) and subsequently embedded in paraffin blocks for further histological examination. Serial sections of 5-6 μm thickness were generated and stained employing a Masson's trichrome staining kit (abs9348, Absin), following the supplier's recommended protocol. Fibrosis was evaluated by imaging four randomly chosen fields from each tissue section, with subsequent measurement of collagen deposition via ImageJ software, expressed as percentage of total area.

### Western blot analysis

Total protein from atrial tissues and cells was isolated with RIPA lysis buffer containing PMSF (ST507, Beyotime) and phosphatase inhibitor, following a previously established protocol 52. The operations were performed on ice. After 30 min of lysis, samples were centrifuged at 12,500 rpm for 25 min. The protein was then boiled and prepared for use. Electrophoretic separation was carried out by SDS-PAGE, followed by transfer to PVDF membranes (7082493, GVS Group, USA). Following blocking with 10% non-fat milk (A600669-0250, Sangon) at 37 °C, the membranes were incubated with primary antibodies overnight at 4 °C. Following TBST washes, they were incubated with secondary antibodies for 2 h at 37 °C. Protein bands were visualized with Tanon imaging system and quantitatively analyzed using ImageJ software. A complete list of antibodies employed in this study is provided in Table S2.

### Immunofluorescence

Atrial tissue sections or cells were fixed by immersion in paraformaldehyde (C104188, Aladdin) for 15 min. Permeabilization was then carried out using Triton X-100 (Sigma) at room temperature. Blocking was achieved by incubating sections with 10% BSA for 2 h at 37 °C. After overnight incubation with primary antibodies at 4 °C, sections were washed and subsequently exposed to appropriate secondary antibodies for 2 h at 37 °C. After DAPI staining (C1002, Beyotime) for nuclear visualization, samples were visualized using a laser scanning confocal microscope (C2 Plus, Nikon). Quantitative analysis of fluorescence intensity was conducted on three randomly selected fields per sample with ImageJ software. The antibodies used are documented in Table S2.

### Detection of ROS and MitoSOX^TM^ Red assays

ROS generation and oxidative stress levels in atrial tissues (6 μm) were evaluated using 10 μM dihydroethidium (DHE, S0063, Beyotime) during 15 min at 37 °C under light-protected conditions. After washing with 0.01 M phosphate-buffered saline (PBS), DHE fluorescence was visualized by confocal microscopy. Atrial sections and cultured cells were incubated with 5 μM MitoSOX™ Red mitochondrial superoxide indicator (M36008, Invitrogen, USA) for 15 min at 37 °C in the dark, followed by DAPI staining to visualize nuclei. Subsequently, all samples were visualized using a confocal microscope.

### Cell culture and treatment

The HL-1 atrial myocyte line (CRL-1446, ATCC; RRID: CVCL_0303) was grown under standard conditions (37 °C, 5% CO₂) in complete DMEM (G4520, Servicebio) medium supplemented with 10% FBS (10100147C, Gibco) and 1% penicillin/streptomycin (15070063, Gibco). Similarly, RAW 264.7 macrophages (TIB-71, ATCC, RRID: CVCL_0493) were maintained under identical culture conditions using the same medium formulation. Throughout the study period, all utilized cell lines were routinely monitored and consistently verified to be free of bacterial, fungal, yeast, viral, and mycoplasma contamination.

For experimental procedures, both cell types were seeded onto 12-well plates, transwell chambers, or confocal dishes upon reaching 60-70% confluence. To establish an *in vitro* model of diabetic metabolic conditions, Cells were treated with a culture medium containing 55 mM glucose and 300 μM palmitic acid (HG + PA, P0500, Sigma), which mimics the combined hyperglycemic and hyperlipidemic milieu observed in diabetes mellitus 4. To further investigate the extent of mitochondrial damage induced by HG + PA, we employed the specific mitochondrial antioxidant mito-TEMPO (CAS 1569257-94-8, Santa Cruz, USA) in cellular study 60. Following respective treatments, cells were processed for protein extraction or subsequent experimental analyses.

### Cell transfection

Short interfering RNA (siRNA) for *Sting* gene knockdown, plasmid for *Sting* gene overexpression, and corresponding negative controls were supplied by Hanbio Co., Ltd (Shanghai, China). HL-1 atrial myocytes were plated in 6-well plates and monitored until achieving 60-70% confluency. Plasmid transfection was carried out with Lipofectamine 3000 reagent (L3000015, Invitrogen) according to standard procedures 56.

### Cell viability assay

HL-1 cell viability was assessed by employing a Cell Counting Kit (CCK-8, C0037, Beyotime Biotechnology, China). Prior to assessment of proliferation, HL-1 cells were seeded at 5 × 10³ cells/well in 96-well plates and treated with PA (100-800 μM) in high glucose (55 mM) medium. After treatment, CCK-8 reagent (10 μL/well) was applied and plates were maintained at 37 °C for 2.5 h, with subsequent measurement of OD450 values on a Multiskan FC microplate reader (Thermo Fisher Scientific).

### Transwell assay

To investigate the crosstalk between atrial myocytes and macrophages under controlled conditions *in vitro*, we established a non-contact co-culture system using HL-1 and RAW 264.7. On day 1, HL-1 cells and RAW 264.7 cells were separately seeded in the upper and lower chambers of 12-well Transwell plates (0.4 μm pore size, 725301, NEST). The following day, HL-1 cells were labeled with 5-ethynyl-2'-deoxyuridine-488 (EdU-488, 10 μM, C0071S, Beyotime) for 24 h to enable subsequent DNA tracking. On day 3, after washing with PBS, the upper chamber containing atrial myocytes received either HG + PA or mito-Tempo treatment. After 24 hours, the upper chamber was washed again and co-cultured with RAW 264.7 cells in the lower chamber for 24 h. This experimental setup ensures physical separation between the two cell types while allowing paracrine communication. After treatment, cells were harvested for subsequent analyses.

### JC-1 staining

Briefly, HL-1 cells cultured in confocal dishes were incubated with the JC-1 reagent (C2003S, Beyotime) at 37 °C for 20 min, followed by washing with 1× JC-1 buffer. The decrease in cell membrane potential can be easily detected by the transition of JC-1 from red (aggregates) to green (monomer) fluorescence. Confocal images were analyzed and quantified according to previously described methods 51.

### Flow cytometry

Atrial tissue samples were minced and digested for 3 h at 37 °C in an enzymatic solution containing collagenase II (1148090, Sigma-Aldrich), DNase I (11284932001, Sigma-Aldrich), and magnesium ions. The resulting cell suspension was filtered and blocked with 2% BSA in PBS. After washing, cells were incubated with antibodies on ice under light-protected conditions. Analysis was performed using a flow cytometer (BD Biosciences), with CD45⁺ CD11b⁺ F4/80⁺ cells identified as macrophages. Data were processed with FlowJo software (TreeStar) following previously established methods 52. A full list of antibodies used is provided in Table S2.

### Statistical analysis

Statistical data are expressed as mean ± SEM. Group comparisons were performed using Student's t-test for two groups, whereas one-way ANOVA followed by Tukey's test was applied for comparisons across multiple groups. Fisher's exact test was applied to analyze atrial fibrillation inducibility. All statistical analyses and graphing were performed with GraphPad Prism (version 10.0, GraphPad). Statistical significance was defined as *p* < 0.05. Non-significant outcomes (*p* > 0.05) are indicated by "ns" in all figures.

## Supplementary Material

Supplementary figures and tables.
