# Supplementary material for: Impairment of mitochondrial quality control exacerbates diabetes-related atrial fibrillation by cGAS-STING signaling pathway and cardiomyocyte-macrophage crosstalk
Source: Theranostics. 2026 Jan 1;16(4):1701–19. doi: 10.7150/thno.124140 (PMC12680528; doi:10.7150/thno.124140)
Supplement: Supplementary file 1 — Supplementary figures and tables. [file thnov16p1701s1.pdf]

Supplementary Material

Impairment of mitochondrial quality control exacerbates diabetes-related atrial fibrillation by cGAS-STING signaling pathway and cardiomyocyte-macrophage crosstalk

Shan Meng[1,2†], Jinfeng Duan[1,3†], Jikai Zhao[1], Zijun Zhou[1], Boxuan Sun[1], Yinli Xu[1], Tao Huang[1], Tao Hong[4,1], Xin Chen[1], Tong Su[1,5], Liming Yu[1]*, Huishan Wang[1]*

[1] State Key Laboratory of Frigid Zone Cardiovascular Disease, Department of Cardiovascular Surgery, General Hospital of Northern Theater Command, 83 Wenhua Road, Shenyang, Liaoning 110016, P. R. China

[2] Department of Anaesthesia, The Sixth Affiliated Hospital, Sun Yat-sen University, Guangzhou, Guangdong 510000, P. R. China.

[3] Postgraduate College, China Medical University, Shenyang, Liaoning 110122, P. R. China

[4] Pediatric Surgery Ward, Fuwai Hospital Chinese Academy of Medical Sciences, ShenZhen 518000, P. R. China

[5] College of Medicine and Biological Information Engineering, Northeastern University, Shenyang, Liaoning 110167, P. R. China

[†] **Shan Meng and Jinfeng Duan contributed equally to this study.**

***Correspondence: Huishan Wang, M.D.** huishanw@126.com or **Liming Yu, M.D.** lmyu2012@163.com.

Figure S1.

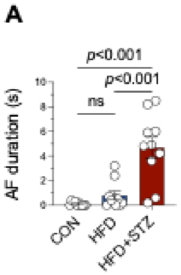

(A) Quantitative analysis of AF duration (n = 10)

Figure S2.

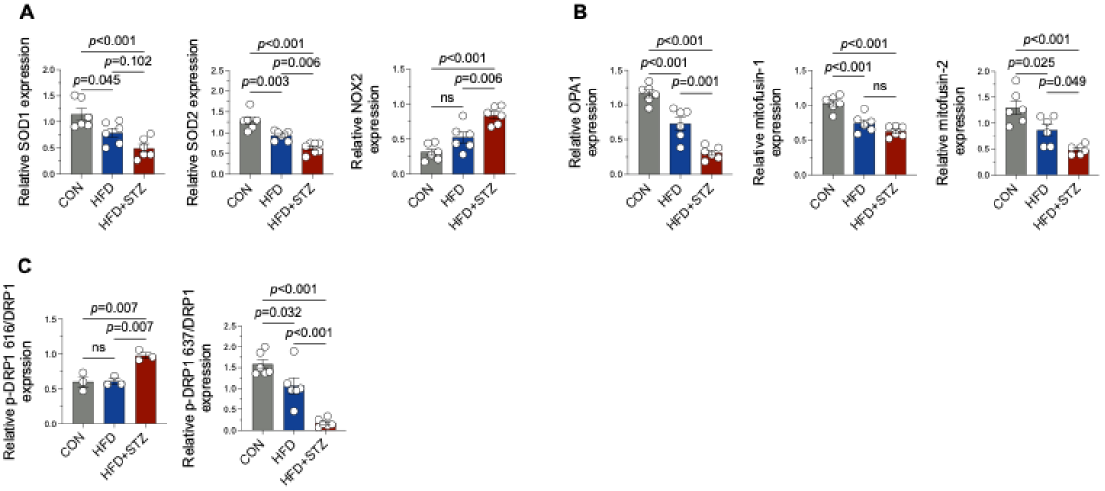

(A) Quantitative analysis of antioxidant enzymes (SOD1, SOD2) and NOX2 expression (n = 6). (B) Mitochondrial fusion proteins quantification (OPA1, MFN1, MFN2; n = 6). (C) Phosphorylation status analysis of DRP1 at Ser616 and Ser637 (n = 6). The p-values were determined by one-way ANOVA followed by Tukey's test, and error bars represent SEM.

Figure S3.

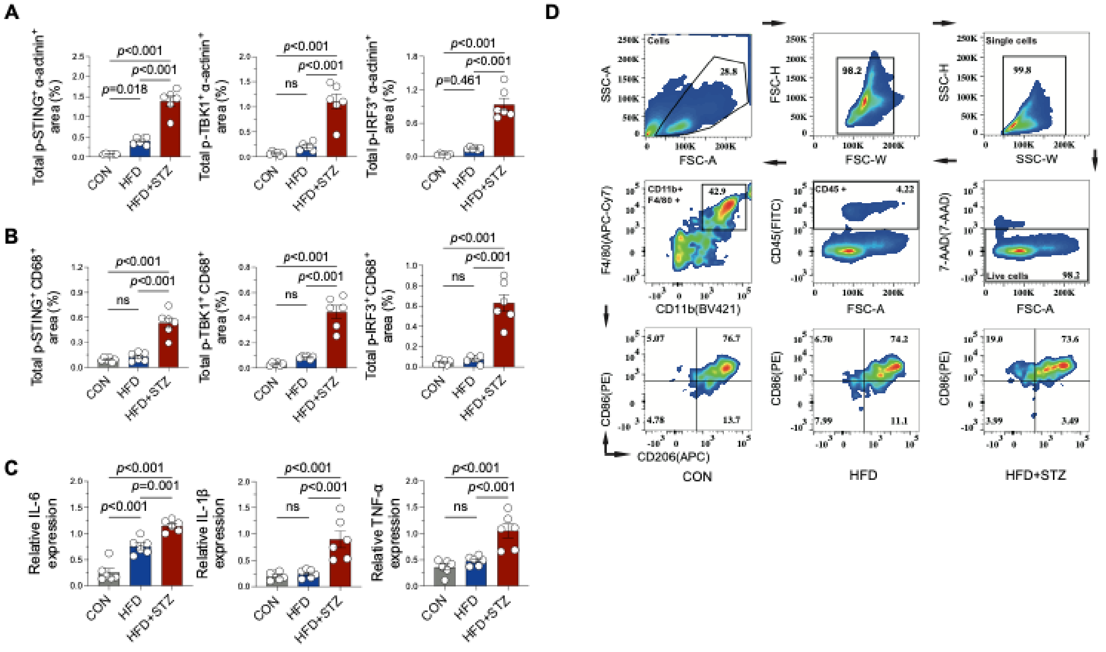

(A) Quantitative analysis of p-STING⁺ α-actinin⁺ area (%) in atrial tissue. (B) Quantitative analysis of p-STING⁺ CD68⁺ area (%) in atrial tissue. (C) Quantitative analysis of IL-6, IL-1β, TNF-α (n = 6). (D) Demonstrative flow cytometry plots of atrial tissue with cells gated based on singlets and immune cell (CD45⁺) subpopulations. Macrophages were identified as CD45⁺, CD11b⁺, F4/80⁺ cells. The p-values were determined by one-way ANOVA followed by Tukey's test, and error bars represent SEM.

Figure S4.

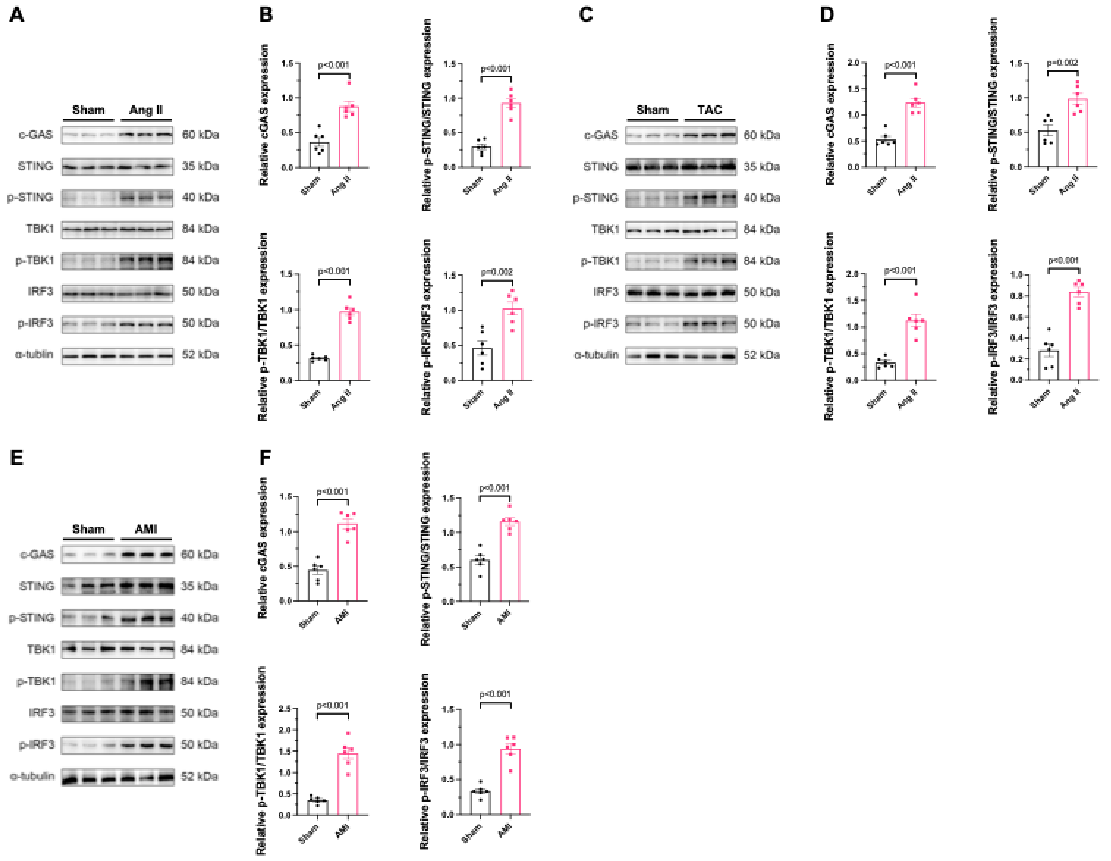

(A-B) Representative Western blots and quantitative analysis of cGAS-STING pathway components (cGAS, p-STING, TBK1, and p-IRF3) in sham and angiotensin II (Ang II)-infused mice (n = 6). (C-D) Representative Western blots and quantitative analysis of cGAS-STING pathway components (cGAS, p-STING, TBK1, and p-IRF3) in in sham and transverse aortic constriction (TAC) mice (n = 6). (E-F) Representative Western blots and quantitative analysis of cGAS-STING pathway components (cGAS, p-STING, TBK1, and p-IRF3) in sham and acute myocardial infarction (AMI) mice (n = 6). The p values were determined by Student’s t test, and error bars represent SEM.

Figure S5.

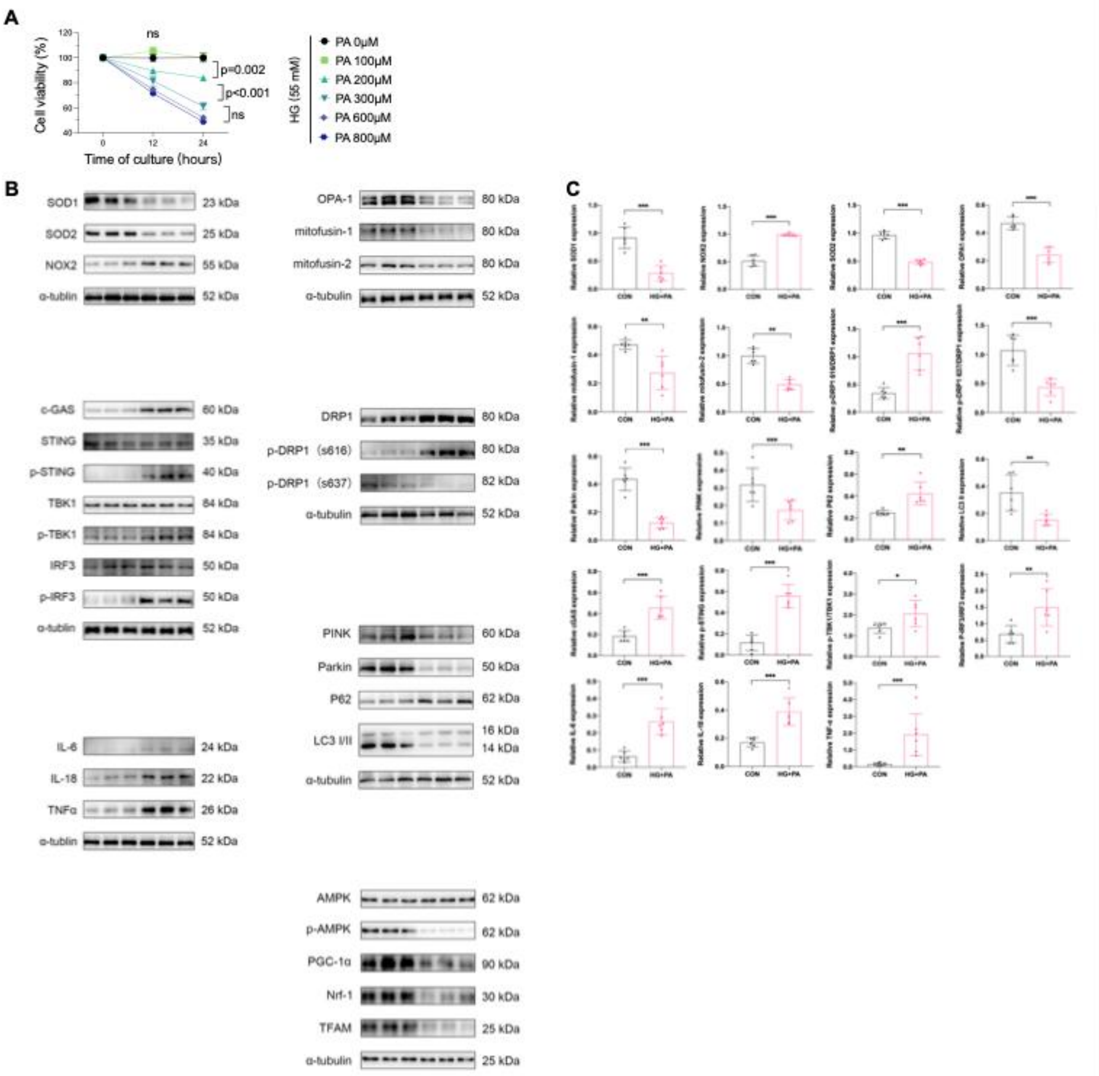

(A) Cell viability (CCK-8 assay) of HL-1 cells treated with HG (55 mM) plus PA (0-800 μM) for 24h. (B-C) Representative western blot images and quantitative analysis of key regulators of oxidative stress, the cGAS-STING signaling pathway, mitochondrial fusion, mitochondrial fission, mitophagy, mitochondrial biogenesis, and proinflammatory cytokines. The p values were determined by Student’s t test, and error bars represent SEM.

Figure S7.

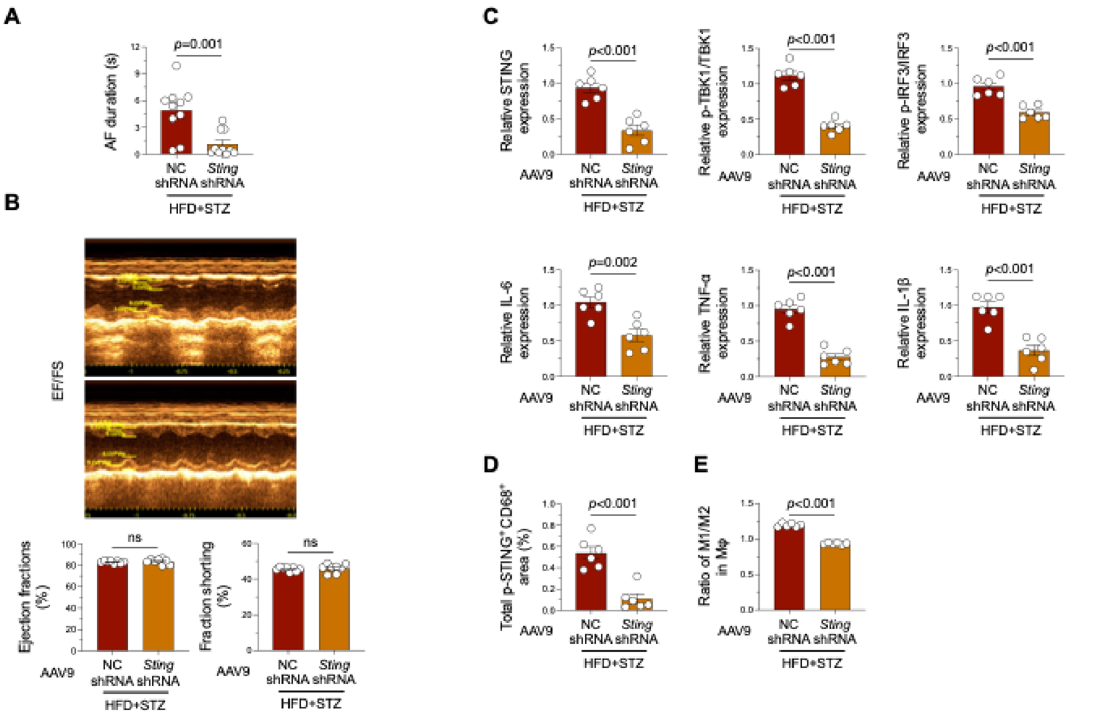

(A) Quantitative analysis of AF duration (n = 10). (B) Echocardiographic assessment of left ventricular function (EF%, FS%; scale bars: 2 mm space, 200 ms time; n = 8). (C) Quantitative analysis of cGAS, p-STING/ STING, p-TBK1/ TBK1, p-IRF3/ IRF3 IL-6, IL-1β and TNF-α (n = 6). (D) Quantitative analysis of p-STING^{+} CD68^{+} area (%) in atrial tissue (n = 6). (E) M1/M2 macrophage ratio by flow cytometry (n = 6). The p values were determined by Student's t test, and error bars represent SEM.

Table S1.

The basic clinical information of included patients

**Characteristics**	**T2DM (n = 4)**	**non-T2DM (n = 4)**	***p* Value**
Age, years	58.00 ± 5.77	37.50 ± 12.26	0.232
Male gender, n (%)	4 (100%)	4 (100%)	> 0.999
Body mass index, kg/m^2	23.01 ± 1.88	24.03 ± 1.96	0.480
Smoke, n (%)	3 (75%)	2 (50%)	> 0.999
Drink, n (%)	3 (75%)	3 (75%)	> 0.999
Left atrial diameter, mm	45.50 ± 9.29	35.25 ± 2.87	0.060
Left ventricular end systolic diameter, mm	47.75 ± 12.53	32.50 ± 1.29	0.052

Left ventricular end diastolic diameter, mm	60.25 ± 9.81	47.25 ± 1.71	0.040
Interventricular septum thickness, mm	9.75 ± 0.96	9.75 ± 0.50	> 0.999
Ejection fraction (%)	0.46 ± 0.17	0.58 ± 0.13	0.125
Fractional shortening (%)	0.24 ± 0.11	0.30 ± 0.02	0.272
Heart valvular disease, n (%)	4 (100%)	1 (25%)	0.142
Hypertension, n (%)	3 (75%)	1 (25%)	0.486

Data presented as mean ± SD or as a ratio. *P* Value determined by Student's *t*-test for continuous variables, and Fisher's exact test for categorical values.

Table S2.

Antibodies Used in This Study (Duplicates Removed)

**Target antigen**	**Vendor or Source**	**Catalog #**	**Working concentration**
Connexin 40	Invitrogen, CA, USA	36-4900	WB: 1:1000, IF: 1:400
α-Actinin	Abcam, Cambridge, MA, USA	ab90421	IF: 1:400
Phospho-STING	Cell Signaling Technology, USA	62912, 50907	IF: 1:400
Phospho-IRF3	Cell Signaling Technology, USA	29047	WB: 1:1000, IF: 1:400
Phospho-TBK1	Cell Signaling Technology, USA	5483	WB: 1:1000, IF: 1:400
CD68/SR-D1	Novus Biologicals, CO, USA	NB600-985	IF: 1:400
CD68	Abcam, Cambridge, MA, USA	ab303565	IF: 1:400
TOMM20	Abcam, Cambridge, MA, USA	ab78547	IF: 1:400
dsDNA	Abcam, Cambridge, MA, USA	ab27156	IF: 1:400
F4/80	Abcam, Cambridge, MA, USA	ab6640	IF: 1:400
α-Tubulin	Cell Signaling Technology, USA	12351s	WB: 1:1000

Connexin 43	Cell Signaling Technology, USA	3512s	WB: 1:1000
SOD1	Santa Cruz, USA	sc-101523	WB: 1:500, IF: 1:400
SOD2	Santa Cruz, USA	sc-137254	WB: 1:500
NOX2	Santa Cruz, USA	sc-130543	WB: 1:500
DRP1	Cell Signaling Technology, USA	8570	WB: 1:1000
Phospho-DRP1 (Ser616)	Cell Signaling Technology, USA	4494	WB: 1:1000
Phospho-DRP1 (Ser637)	Abcam, USA	ab193216	WB: 1:1000
OPA1	Cell Signaling Technology, USA	80471	WB: 1:1000
Mitofusin-1	Cell Signaling Technology, USA	9482	WB: 1:1000
Mitofusin-2	Cell Signaling Technology, USA	14739	WB: 1:1000
SQSTM1/p62	Cell Signaling Technology, USA	23214	WB: 1:1000
Parkin	Cell Signaling Technology, USA	4211	WB: 1:1000
PINK1	Santa Cruz, USA	sc-517353	WB: 1:500
LC3A/B	Cell Signaling Technology, USA	4108	WB: 1:1000
Phospho-AMPKα (Thr172)	Cell Signaling Technology, USA	50081s	WB: 1:1000
AMPKα	Cell Signaling Technology, USA	5831	WB: 1:1000
NRF1	Santa Cruz, USA	sc-28379	WB: 1:500
PGC-1α	Santa Cruz, USA	sc-518025	WB: 1:500
Tfam	Santa Cruz, USA	sc-166965	WB: 1:500
cGAS	Cell Signaling Technology, USA	31659, 15102	WB: 1:1000
STING	Cell Signaling Technology, USA	13647	WB: 1:1000
STING	Proteintech Group, USA	19851-1-AP	WB: 1:1000
Phospho-STING	Cell Signaling Technology, USA	72971, 50907	WB: 1:1000
IRF3	Santa Cruz, USA	sc-33641	WB: 1:500
TBK1	Cell Signaling Technology, USA	38066s	WB: 1:1000

IL-6	Cell Signaling Technology, USA	12912	WB: 1:1000
IL-1β	Santa Cruz, USA	sc-12742	WB: 1:500
TNF-α	Santa Cruz, USA	sc-52746	WB: 1:500
Myc tag	Beyotime Biotech, China	af0033	WB: 1:500
Goat anti-rabbit IgG	Zhong Shan-Golden Bridge, China	zb2301	WB: 1:2000
Goat anti-mouse IgG	Zhong Shan-Golden Bridge, China	zb2305	WB: 1:2000
Alexa Fluor 488 donkey anti-mouse IgG (H + L)	Invitrogen, CA, USA	A32766	IF: 1:300
Alexa Fluor 568 donkey anti-rabbit IgG (H + L)	Invitrogen, CA, USA	A10042	IF: 1:300
CD45-FITC	BioLegend	103108	1:200
CD11b-BV421	BioLegend	101235	1:200
F4/80-APC-Cy7	BioLegend	123118	1:200
CD86-PE	eBioscience	12-0862-82	1:200
CD206-APC	eBioscience	17-2061-82	1:200
7-AAD viability dye	eBioscience	00-6993-50	1:200
